# Impact on birth weight and child growth of Participatory Learning and Action women’s groups with and without transfers of food or cash during pregnancy: Findings of the low birth weight South Asia cluster-randomised controlled trial (LBWSAT) in Nepal

**DOI:** 10.1371/journal.pone.0194064

**Published:** 2018-05-09

**Authors:** Naomi M. Saville, Bhim P. Shrestha, Sarah Style, Helen Harris-Fry, B. James Beard, Aman Sen, Sonali Jha, Anjana Rai, Vikas Paudel, Raghbendra Sah, Puskar Paudel, Andrew Copas, Bishnu Bhandari, Rishi Neupane, Joanna Morrison, Lu Gram, Anni-Maria Pulkki-Brännström, Jolene Skordis-Worrall, Machhindra Basnet, Saskia de Pee, Andrew Hall, Jayne Harthan, Meelan Thondoo, Sonja Klingberg, Janice Messick, Dharma S. Manandhar, David Osrin, Anthony Costello

**Affiliations:** 1 Institute for Global Health, University College London, London, United Kingdom; 2 Mother and Infant Research Activities (MIRA), Kathmandu, Nepal; 3 MRC Clinical Trials Unit, University College London, London, United Kingdom; 4 UN World Food Programme, Rome, Italy; 5 Save the Children UK, London, United Kingdom; TNO, NETHERLANDS

## Abstract

**Background:**

Undernutrition during pregnancy leads to low birthweight, poor growth and inter-generational undernutrition. We did a non-blinded cluster-randomised controlled trial in the plains districts of Dhanusha and Mahottari, Nepal to assess the impact on birthweight and weight-for-age z-scores among children aged 0–16 months of community-based participatory learning and action (PLA) women’s groups, with and without food or cash transfers to pregnant women.

**Methods:**

We randomly allocated 20 clusters per arm to four arms (average population/cluster = 6150). All consenting married women aged 10–49 years, who had not had tubal ligation and whose husbands had not had vasectomy, were monitored for missed menses. Between 29 Dec 2013 and 28 Feb 2015 we recruited 25,092 pregnant women to surveillance and interventions: PLA alone (n = 5626); PLA plus food (10 kg/month of fortified wheat-soya ‘Super Cereal’, n = 6884); PLA plus cash (NPR750≈US$7.5/month, n = 7272); control (existing government programmes, n = 5310). 539 PLA groups discussed and implemented strategies to improve low birthweight, nutrition in pregnancy and hand washing. Primary outcomes were birthweight within 72 hours of delivery and weight-for-age z-scores at endline (age 0–16 months). Only children born to permanent residents between 4 June 2014 and 20 June 2015 were eligible for intention to treat analyses (n = 10936), while in-migrating women and children born before interventions had been running for 16 weeks were excluded. Trial status: completed.

**Results:**

In PLA plus food/cash arms, 94–97% of pregnant women attended groups and received a mean of four transfers over their pregnancies. In the PLA only arm, 49% of pregnant women attended groups. Due to unrest, the response rate for birthweight was low at 22% (n = 2087), but response rate for endline nutritional and dietary measures exceeded 83% (n = 9242). Compared to the control arm (n = 464), mean birthweight was significantly higher in the PLA plus food arm by 78·0 g (95% CI 13·9, 142·0; n = 626) and not significantly higher in PLA only and PLA plus cash arms by 28·9 g (95% CI -37·7, 95·4; n = 488) and 50·5 g (95% CI -15·0, 116·1; n = 509) respectively. Mean weight-for-age z-scores of children aged 0–16 months (average age 9 months) sampled cross-sectionally at endpoint, were not significantly different from those in the control arm (n = 2091). Differences in weight for-age z-score were as follows: PLA only -0·026 (95% CI -0·117, 0·065; n = 2095); PLA plus cash -0·045 (95% CI -0·133, 0·044; n = 2545); PLA plus food -0·033 (95% CI -0·121, 0·056; n = 2507). Amongst many secondary outcomes tested, compared with control, more institutional deliveries (OR: 1.46 95% CI 1.03, 2.06; n = 2651) and less colostrum discarding (OR:0.71 95% CI 0.54, 0.93; n = 2548) were found in the PLA plus food arm but not in PLA alone or in PLA plus cash arms.

**Interpretation:**

Food supplements in pregnancy with PLA women’s groups increased birthweight more than PLA plus cash or PLA alone but differences were not sustained. Nutrition interventions throughout the thousand-day period are recommended.

**Trial registration:**

ISRCTN75964374.

## Introduction

Low birthweight (LBW: <2500 g), of infants small-for-gestational-age (SGA) or preterm, increases the risks of neonatal mortality [1, 2] and infant mortality [3], poor cognitive development [4], stunting [5], and lifelong susceptibility to non-communicable diseases [6, 7]. LBW remains a major public health problem in low-income settings, particularly in South Asia [8] and the WHO has set a target to reduce LBW by 30% by 2025 [9]. The urgency of addressing intergenerational undernutrition by intervening prenatally or during pregnancy to decrease SGA and stunting at birth is acknowledged [5, 8, 10], but our understanding of effective means of doing so is limited. Renewed WHO interest in low birth weight as an indicator for tracking country health [9] means use of birthweight as an outcome (as opposed to preterm and SGA) is of particular importance.

We reviewed evidence on three types of pregnancy-focused intervention that might improve birthweight: food supplements, cash transfers, and behaviour change communications. To review the effect of food supplements during pregnancy on child nutritional status we drew upon recent meta-analyses of trials of food supplementation, which all demonstrated a significant increase in birthweight [11–14]. Gresham 2014 found a raw mean difference (RMD) of 125 g from food supplementation or 94 g in studies limited to low or middle income countries (LMICs) and undernourished women [11], Imdad and Bhutta 2012 73 g [12], Ota 2012 41 g, (47, 77) [14] and Stevens 2015 found a standardised mean difference of 0.2 (0.03–0.38) (weight difference in grams not given) [13]. While updating the search of Stevens 2015, which focused on LMICs, we identified a Cambodian study of supplementation with a corn-soy blended food in pregnancy, which showed no differences in anthropometry [15]. Recent trials of Lipid-based Nutrient Supplements (LNS) in pregnancy have shown inconsistent results. Increases in birthweight of 85 g were demonstrated in Ghana [16], and of 41 g in Bangladesh [17], but no differences in anthropometry were shown in Malawi (either at birth or 18 months) [18].

Girard’s 2012 review of the effects of nutrition education and counselling (NEC) on birth weight showed an increase of 105 g (18, 193) overall and 152 g (-81, 384) in LMICs, but when disaggregated by whether the NEC was combined with food or micronutrient supplements, only the trials of NEC combined with supplements significantly increased birthweight whilst NEC alone did not: mean difference -8 g (-87, 70) for NEC alone and 0.1 g (0.0, 0.3) with NEC plus additional messages (16). Similarly, Ota’s review showed a significant birthweight increase of 490 g, (428, 552) from 2 trials of nutritional education alone with low quality evidence, and no increase in one trial with adequately nourished women 15 g (-76, 106) [14]. Quality of evidence around NEC and birth outcomes was generally low [19].

To review evidence on cash transfers during pregnancy on birthweight, we conducted searches in PubMed, Embase, The Cochrane Library, African Index Medicus, Web of Science and the Reproductive Health library using the inception date and December 2016 as inclusion dates, with no language restriction. For the effect of cash transfers on birthweight or child nutrition we used the search terms “cash transfer”, pregnant OR pregnancy, AND birthweight OR “child nutrition” respectively, without search limitations. Studies were included if they were original peer-reviewed articles or systematic reviews describing studies in which cash transfers were targeted to pregnant women in LMICs. Studies were excluded if they did not include either birthweight or child nutrition as one of the outcomes. Limited evidence of the effect of cash transfers on birthweight comes from large-scale programmes of cash transfers and income supplements in Mexico and Uruguay, which show increases in birthweight of the order of 127 g [20] and 31 g [21] respectively. In Mexico, Rivera (2004) found that children at young ages (0–6 months) in Progresa incentive-based intervention areas were 1.1 cm longer [22]. Leroy’s review of conditional cash transfers on child nutrition found a 1.53 cm length gain (0.41 Z-scores) but little impact upon micronutrient status [23]. We found no publications comparing effects of food supplements versus cash transfers on birthweight.

To review the effect of behaviour change interventions on birthweight we used the databases and inclusion/ exclusion criteria listed above, with the search terms “Child OR infant AND weight-for-age OR length-for-age OR nutritional status OR weight OR length OR stunting OR wasting OR underweight OR z score and pregnan* combined with either [women's group* OR participatory learning* OR community mobilization OR community participation] OR [nutrition education OR nutrition counseling OR “nutrition education and counseling” OR nutrition information]”.

We found no publications evaluating the effects on birthweight or child nutrition of Participatory Learning and Action (PLA) or community mobilization, apart from a study by Nair et al in India which found no significant effects on child stunting or birthweight but lower odds of underweight at 18 months of age (adjusted OR: 0.81 (0.66, 0.99)) [24, 25]. However we found that nutrition education interventions using participatory community-based approaches focused on practical behaviour were more likely to be effective than knowledge transfer alone [26]. Moreover, PLA with women’s groups was effective in reducing other health outcomes, including neonatal and maternal mortality in rural communities [27], and was pro-poor in coverage [28] and impact [29], and may influence nutrition-related behaviours [30] and dietary diversity [31].

We hypothesised that an improved behaviour change strategy using Participatory Learning and Action (PLA) women’s groups combined with transfers of food or cash to pregnant women would lead to increases in birthweight and child weight-for-age z-score in the first 16 months. We did a cluster-randomised controlled trial to compare with usual government programmes the effects on birthweight and child nutritional status of three interventions—PLA alone, PLA plus food transfers, and PLA plus cash transfers. We also assessed improvements in maternal nutritional status, eating behaviour in pregnancy, feeding behaviours, and institutional delivery.

## Materials and methods

### Study design

We did a four-arm cluster-randomised controlled trial with 20 clusters randomly allocated to each arm, in Dhanusha and Mahottari districts in the central low-lying plains (Terai) of Nepal. Detailed descriptions of the study are available in the protocol [32] and a paper describing the electronic data collection system [33]. The population of 1.4 million [34] live mostly in villages and agricultural livelihoods predominate. Markets selling local produce run frequently, within easy reach of most communities. Undernutrition of women is common: in 2011, 39% were underweight (BMI <18.5 kg/m^2^), 14% had low stature (<145 cm) [35], and low birthweight prevalence exceeded 28% [36]. The population has strong patriarchal and caste traditions. Since 2007, the area has suffered severe intermittent unrest associated with Terai-based (Madhesi) groups mobilising to improve political representation for plains people.

Ethical approval was obtained from the Nepal Health Research Council (108/2012) and the University College London (UCL) Ethical Review Committee (4198/001).

### Participants

Married women aged 10–49 years, who had not had tubal ligation and whose husbands had not had vasectomy, were eligible for inclusion. Eligible women were enrolled when a study enumerator identified them as having missed two consecutive menses, with a positive pregnancy test or obviously pregnant appearance. They were issued with a Quick Response (QR) coded photo ID card any time after eight weeks gestation. We obtained written consent from women for menstrual monitoring and trial follow-up at enrolment. For married girls who were minors (< = 17 years) guardians were involved fully in the consent process and gave oral consent while the teenage participant gave written consent. Consent for inclusion of clusters in the trial was gained from cluster gatekeepers (Village Development Committee (VDC) secretaries) after randomisation and before community entry.

### Randomisation

To reduce heterogeneity, we excluded clusters that had received previous interventions, large towns and municipalities, hilly, forested, or non-Maithili-speaking clusters, clusters along the East-West highway, and clusters with estimated population <4000 or >9200, leaving 80 of 179 clusters for randomisation. We defined four strata of 20 clusters each on the basis of population size (4000–6399 versus 6400–9200) and accessibility during monsoon season, and conducted block randomisation using a ‘tombola method’ with community stakeholders. Randomisation was at the level of the VDC cluster (mean population 6150).

We randomised 80 VDC clusters into four arms (20 per arm): control receiving current Government of Nepal programmes, PLA alone, PLA plus a food supplement, or PLA plus a cash transfer. Concealment of allocation was impossible due to the cluster-level study design. Community meetings were held in July-August 2013 to introduce the study objectives and explain the interventions.

### Interventions

#### Participatory Learning and Action

All intervention arms included a similar behaviour change strategy: Participatory Learning and Action (PLA) with government-mandated women’s groups facilitated by Female Community Health Volunteers (FCHVs). According to Nepal government policy, FCHVs should convene one women’s group per month in their working areas, but in control areas these were inactive or lacked agendas for discussion. Only 29% of FCHVs could read and write with ease and their workload in government health programmes was high. We worked with 539 groups (nine per cluster), revitalising inactive groups (18%). From September 2013, 179 groups met regularly over two years in the PLA arm and 180 groups in each of the PLA plus transfer arms.

We enlisted literate ‘nutrition mobilisers’ to assist with group facilitation, transfer distribution, and record-keeping. Both mobilisers and FCHVs were paid a monthly incentive (NPR 800 ≈ USD 8) to facilitate women’s groups and attend orientation sessions to review previous meetings and discuss topics for the next. Thirty supervisors, educated to School Leaving Certificate or above, were employed to support the 540 FCHVs and 539 mobilisers.

Groups followed a pictorial manual with simple Nepali text, over 20 meetings in four phases: learning about the problem of LBW and malnutrition in pregnancy, formulation of strategies to overcome barriers to improved health and nutrition during pregnancy, implementation of strategies, and evaluation. Strategy implementation began in July-August 2014 and included home visits to pregnant women who did not attend groups, showing picture cards in the community, separate meetings with mothers-in-law, adolescent girls, or male family members, rallies on the importance of maternal nutrition, and screening pregnancy-related videos. Although we designed a counselling manual for nutrition mobilisers to undertake structured home visits to pregnant women, in practice mobilisers were too overloaded to undertake intensive home visits, so the counselling manual was not used and home visits were limited.

In the control arm FCHVs delivered usual government outreach services (family planning, immunisation, vitamin A and iron-folate distribution, health promotion, and so on) and were required to run a monthly mothers group meeting. FCHVs set their own agendas for these meetings, using flip charts provided by the District Public Health Office. A substudy demonstrated that these groups we not very active, except in areas where another NGO was / or had recently been working with the FCHVs to run the groups.

#### Cash and food transfers

The food transfer in the PLA plus food arm was 10 kg per month of a fortified balanced energy protein (BEP) supplement of wheat-soya blended flour with 10% added sugar called Super Cereal (previously WSB+). The Super Cereal ration was sufficient for 150 g for the pregnant woman and 180 g for family sharing per day. The recommended 150 g daily intake provided close to the UNIMMAP micronutrient supplement’s dose of micronutrients, in addition to 600 kcal and >20 g of protein. Full details of the macro- and micronutrient composition are provided in the trial protocol [32]. When supplementing the usual diet, this was expected to cover the nutritional needs of the pregnant woman, especially if combined with consumption of the standard iron-folate supplement distributed to all pregnant women by Government of Nepal Ministry of Health. The cash transfer in the PLA plus cash arm was NPR 750 (≈USD7.5) per month, equivalent to the cost of 10 kg of Super Cereal or two days’ wage labour.

Women were entitled to one monthly transfer, up to a maximum of seven transfers until delivery, provided they received it personally and had their photo ID card. Family members were not permitted to receive transfers on her behalf, but women could receive transfers through home visits if they were physically unable to attend groups. Receipt of transfers was recorded on thumb-printed or signed paper vouchers.

Interventions were monitored using questionnaires on smartphones. Supervisors used observation checklists when attending women’s group meetings, community planning meetings, strategy implementation, and participatory evaluation. Mobilisers recorded group attendance, transfer distribution, and home visits. Qualitative focus groups and semi-structured interviews were conducted with trial participants and supervisors to explore implementation and impact mechanisms. Monthly narrative observation of review meetings and of one women’s group per arm also enabled description of intervention implementation.

### Benefits to non-transfer arms

Training in maternal nutrition was provided to health workers from all study areas regardless of allocation. Women in control or PLA only arms received NPR 1000 (≈ USD 10) at the end of the study. All women whose children were measured at endpoint received two bars of soap and a cotton towel.

### Outcomes

Primary outcomes were birthweight measured within 72 hours and weight-for-age z-score (WAZ) measured cross-sectionally in children aged 0–16 months (S1 Table). The WAZ outcome was added in February 2015 in response to problems capturing sufficient birthweight measures that resulted from ethnic conflict within the study team, and due to lack of funds to continue birthweight measurement. Secondary outcomes included length and head circumference at birth, measured within 10 days, prevalence of LBW within 72 hours, eating behaviour and weight during pregnancy, maternal and newborn morbidity, preterm delivery, miscarriage, stillbirth, and neonatal mortality, maternal death and institutional delivery. Additional secondary outcomes measured at 0–16 months included length-for-age (LAZ) and weight-for-length (WLZ) z-scores, head circumference, maternal body mass index (BMI) and mid-upper arm circumference (MUAC), breastfeeding and infant and young child feeding, and child morbidity. Weight taken within 10 days of birth, as used in a recent trial in Mumbai [37], and WAZ within 42 days were defined as additional secondary outcomes. Exposures to women’s groups, food or cash transfers, home visits, and group interventions in the community were measured at endpoint.

### Data collection

Enumerators maintained menstrual monitoring registers to track missed menses, pregnancies, births, women’s vital status, and migration. Enumerators informed interviewers by text messages about pregnancies and births. After confirmation of pregnancy using urine dip test, VDC interviewers completed an interview about socioeconomic status and past pregnancies, and administered follow-up questionnaires at home: (i) early pregnancy (8–30 weeks), (ii) late pregnancy (31 weeks to birth), (iii) birth (0–42 days), (iv) post-neonatal (43–84 days). Further questionnaires for (v) mother and (vi) child were completed cross-sectionally during the endpoint follow-up at ward-level data collection points. Data were entered on Android smartphones running CommCare (www.commcarehq.org) (i-iv), and ODK Collect (www.opendatakit.org) (v-vi) [33].

Anthropometric measures were collected using Tanita BD590 scales with precision of 10 g for children, Tanita solar weighing scales with precision of 100 g for mothers, “Shorr boards” with precision of 1 mm for height and length, and Seca 212 circumference tapes for MUAC and head circumference. Mother’s weight and MUAC were measured in early pregnancy, late pregnancy, postpartum and at endpoint follow-up. Height was measured in early pregnancy and at endpoint follow-up as needed. Child weight, length and head circumference were measured at birth (ideally within 72 hours of delivery), at 6–8 weeks, and at endpoint follow-up. Duplicate measurements were taken routinely and a third measurement taken when differences between the first two exceeded acceptable limits. Standardisation tests were done on three occasions. Intra- and Inter-observer Technical Error of Measurement (TEM) generally met targets, with head circumference and MUAC being the most difficult measures to standardize. Poorly performing data collectors received additional training and support.

### Sample size

At the trial design stage, we calculated that with birthweight as primary outcome, assuming a standard deviation (SD) of 410 g based on previous data, an intra-cluster correlation coefficient (ICC) of 0.01, and two-tailed 5% significance level, the study would have 80% power to demonstrate a difference of 50 g between two study arms if we obtained 163 birthweights per cluster from at least 17 clusters per arm. Due to difficulties obtaining birthweights, but seeing little chance of cluster dropout, we repeated the calculation during the trial. With all 80 clusters contributing data, a revised target of 111 birth weights per cluster, 2220 per arm and 8880 in total, provided the same power. Sample size targets related to birthweights obtained within 72 hours from resident participants who could have had >16 weeks exposure to interventions. For WAZ, we aimed to measure 150 eligible children per cluster. Assuming an ICC of 0.01 and SD of 1, this would have 84% power to demonstrate a difference of 0.12 between two arms.

### Statistical methods

For analysis we defined two types of participant. Women who enrolled in the census or were newlywed in-migrators living in their husband’s families were ‘trial participants’. They were eligible for inclusion in primary intention-to-treat analyses if they delivered after interventions had been running for >16 weeks (after 4 June 2014) and before the start of the endpoint follow-up (20 June 2015). Women who were not enrolled during the census or who in-migrated to their parental or ‘other’ home, and women who delivered outside the eligible period, were ‘intervention participants’. They received interventions as allocated, but were not included in analyses.

Multiple births, stillbirths, neonatal deaths occurring before birthweight was taken, infants with congenital abnormalities, and children whose mothers died were ineligible for the analysis of primary outcomes. For women who were eligible and delivered, miscarried, or terminated pregnancies twice in the study period, we included the outcomes of both pregnancies in analysis of the birthweight primary outcome and other surveillance outcomes. At endpoint follow-up, we obtained anthropometric measurements from one child per woman. For women who had two pregnancies in the study, we took measurements for the youngest child where possible. We excluded any anthropometric data that were outside the WHO 2006 exclusion criteria for high or low z-scores [38]. At endpoint this amounted to 73 LAZ (0.60%), 21 WLZ (0.17%) and 30 WAZ (0.25%) measures. At delivery excluded z scores amounted to 6 LAZ (0.06%), 23 (0.22%) WHZ and 0 WAZ measures.

Primary analyses were intention-to-treat, analysed by allocation regardless of exposure or gestational age at enrolment. We reported mean differences between arms for continuous outcomes and odds ratios for binary outcomes. These were calculated using linear, logistic, or ordered logistic regression models as appropriate, and presented with 95% confidence intervals. All analyses, including effect measures and summary statistics, were based on individual rather than cluster-level data. Regression analyses included a random effect for cluster, and all clusters were included in analyses unless otherwise stated.

Primary comparisons were between each of the three intervention arms and the control arm. In the event that the PLA plus cash or the PLA plus food arm was significantly superior to the control arm, a secondary comparison was made with the PLA arm to assess the impact of adding cash or food. We adjusted for randomisation stratum, age of child (for anthropometric outcomes), for a base set of covariates that could be predictive of the primary outcomes, and for further covariates for which an imbalance was seen between arms. The base set included child sex, maternal age, education, gravidity, household wealth score, and caste/religious group. We adjusted for gravidity and maternal age through a single covariate with three categories: adolescent mother, non-adolescent primigravid mother, and non-adolescent parous mother.

We conducted subgroup analyses on WAZ and LAZ using data from the endpoint follow-up. Exposure in the PLA arm was defined by the number of meetings attended, grouped into thirds. For transfer arms, exposure was defined by the number of transfers of food or cash received, grouped into thirds. We present effect measures for exposure subgroups relative to all control participants. Formal testing for dose-response was conducted for each intervention arm, using data from that arm alone, based on a regression model using the continuous variable for exposure and adjusting for covariates as in the primary analysis.

Three socioeconomic status subgroups were defined using the multi-dimensional poverty index (MPI) [39]. The MPI was calculated with the nutritional dimension and ownership of inexpensive assets removed, in case these were affected by the interventions. We tested whether the effect of each intervention differed across MPI subgroups by testing the interaction in a regression model adjusting for covariates as in the primary analysis.

Analysis of endpoint WAZ was conducted with and without multiple imputation of missing covariate values. The missing values were imputed using the chained equations method (function ‘mi’ in Stata) using information from the observed covariates, randomisation arm, and WAZ, ignoring geographical clustering in the data. Analysis after imputation is considered primary. Analyses for all other outcomes were based on available cases only. We pre-specified that we would formally analyse intervention effects on birthweight within 72 hours, however complete these data were. Other outcomes were only formally analysed if the data were >40% complete overall, and the proportion of missing data did not vary severely across arms. Otherwise, only summary statistics are reported by arm. Analyses were conducted in Stata 14. The trial statistician was blinded to allocation.

A Data Monitoring Committee (DMC) and a Trial Steering Committee oversaw the study.

Both reviewed the protocol and the DMC approved the trial analysis plan. Some additional analyses were conducted at the request of reviewers of this article.

Measurement of birthweight within 72 hours was more challenging than we anticipated, a situation that was severely exacerbated by ethnic conflict within the field team. Also, funds were not available to extend the study, which made it impossible to collect sufficient birthweight measurements to reach the target sample size. In response to this, the study team, in consultation with the Trial Steering Committee in February 2015, decided to add the WAZ primary outcome and selected secondary outcomes (collected cross-sectionally at endpoint) and to stop enrolment of pregnancies on 28 February 2015, before the target sample size for birth weights could have been achieved.

Our study protocol [32], available as supporting information S1 File, listed the trial outcomes and indicated the time points they were measured. In an effort to reduce the number of outcomes tested, minor modifications to the list of secondary outcomes to be analysed were made when preparing the analysis plan on the basis of capture rates, but not using any comparisons between study arms. The analysis plan (which was approved by the DMC) specified time points of measurements and which outcomes should and should not be formally analysed. Summary statistics by study arm are provided for all outcomes listed in the analysis plan in our results tables. Some secondary outcomes with a capture rate below 40% of eligible mothers / children were analysed on the request of reviewers of this article. These include LBW within 72 hours, weight, length and head circumference within 10 days, weight-for-age Z score within 42 days, and mother’s weight in late pregnancy, dietary diversity score in early or late pregnancy, number of eating occasions per day during pregnancy, and neonatal morbidity (7 danger signs).

Mortality outcomes and miscarriages were not formally analysed because lack of resources prevented a comprehensive endpoint census of outcomes of all enrolled pregnancies. At endpoint, participating women reported for anthropometric measurement at a village data collection point, which may have missed women who miscarried or whose babies had died. Notable changes between the published study protocol and the final analysis plan were addition of birthweight within 10 days, weight-for-age z score in the first 42 days, length within 10 days (instead of length for age Z-score), mother’s weight in late pregnancy (rather than weight gain), gestational age at delivery, and consumption of key food groups (animal, green leafy vegetable and combined fruits and vegetables) by children and post-neonatal mortality at endpoint (though the latter was not analysed for reasons given above). For comparison with other studies and ease of interpretation, we have also added summaries (but no analyses) of stunting, wasting, underweight (<-2 z scores for length-for-age, weight-for-length and weight-for-age respectively calculated using the WHO 2006 growth standard) and a binary indicator of maternal adequate dietary diversity (5 or more food groups).

The trial consort checklist, a ReadMe file describing the trial dataset and a data use application form are available as supporting information in S2 File, S3 File and S4 File respectively.

Trial Registration number: ISRCTN75964374.

## Results

**Fig 1:**
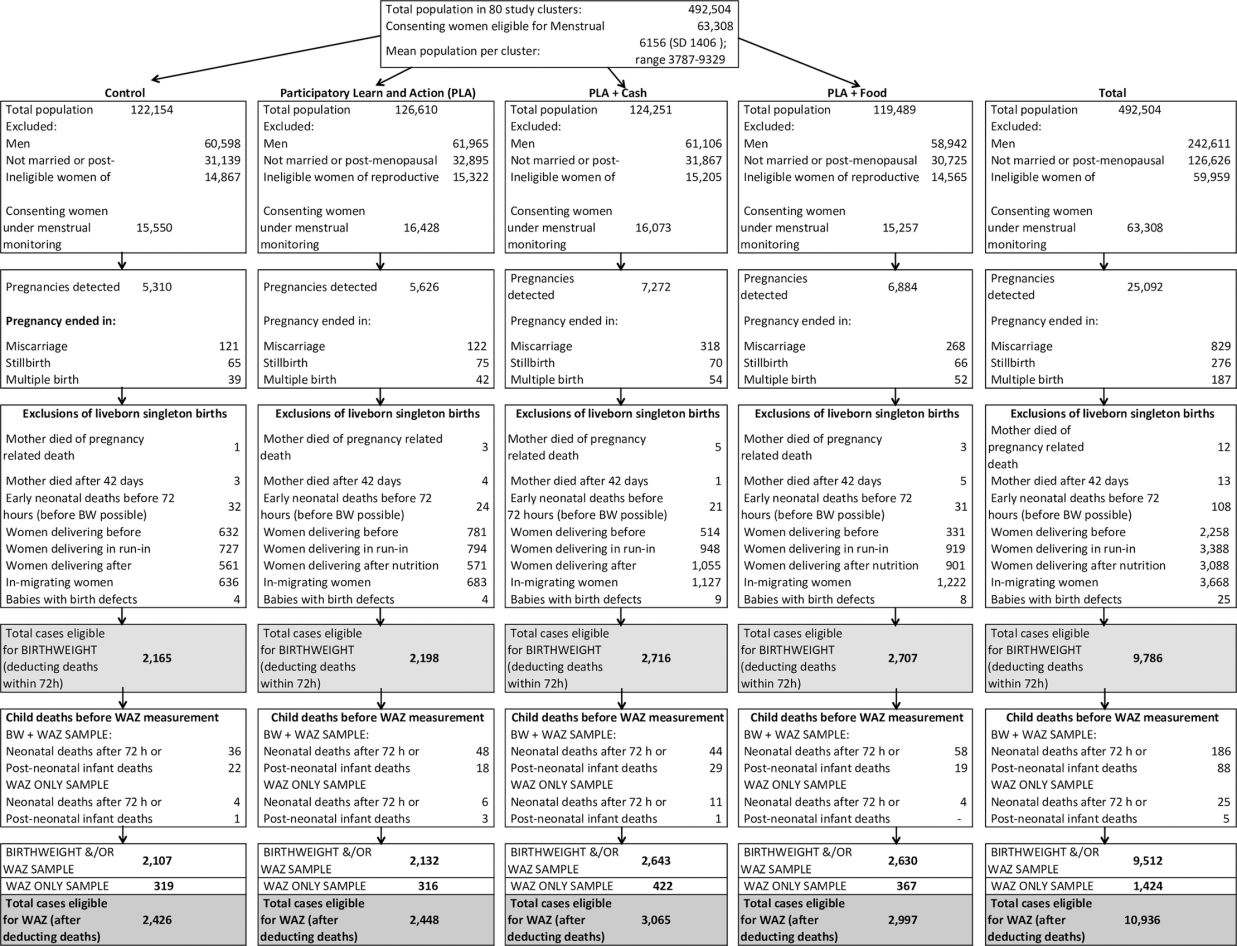
Participant flow.

All dates of recruitment, follow-up and intervention implementation are provided in Fig 1 and the trial profile provides full details of participant flow (Fig 1). From 63,308 women who consented to menstrual monitoring, we recruited 25,092 pregnancies into interventions and surveillance, and after exclusions 10,936 permanently resident women who delivered between 4 June 2014 and 20 June 2015 were eligible for (WAZ) outcome analyses (control: n = 2426; PLA alone n = 2448; PLA plus cash n = 3065; PLA plus food n = 2997).

**Fig 2:**
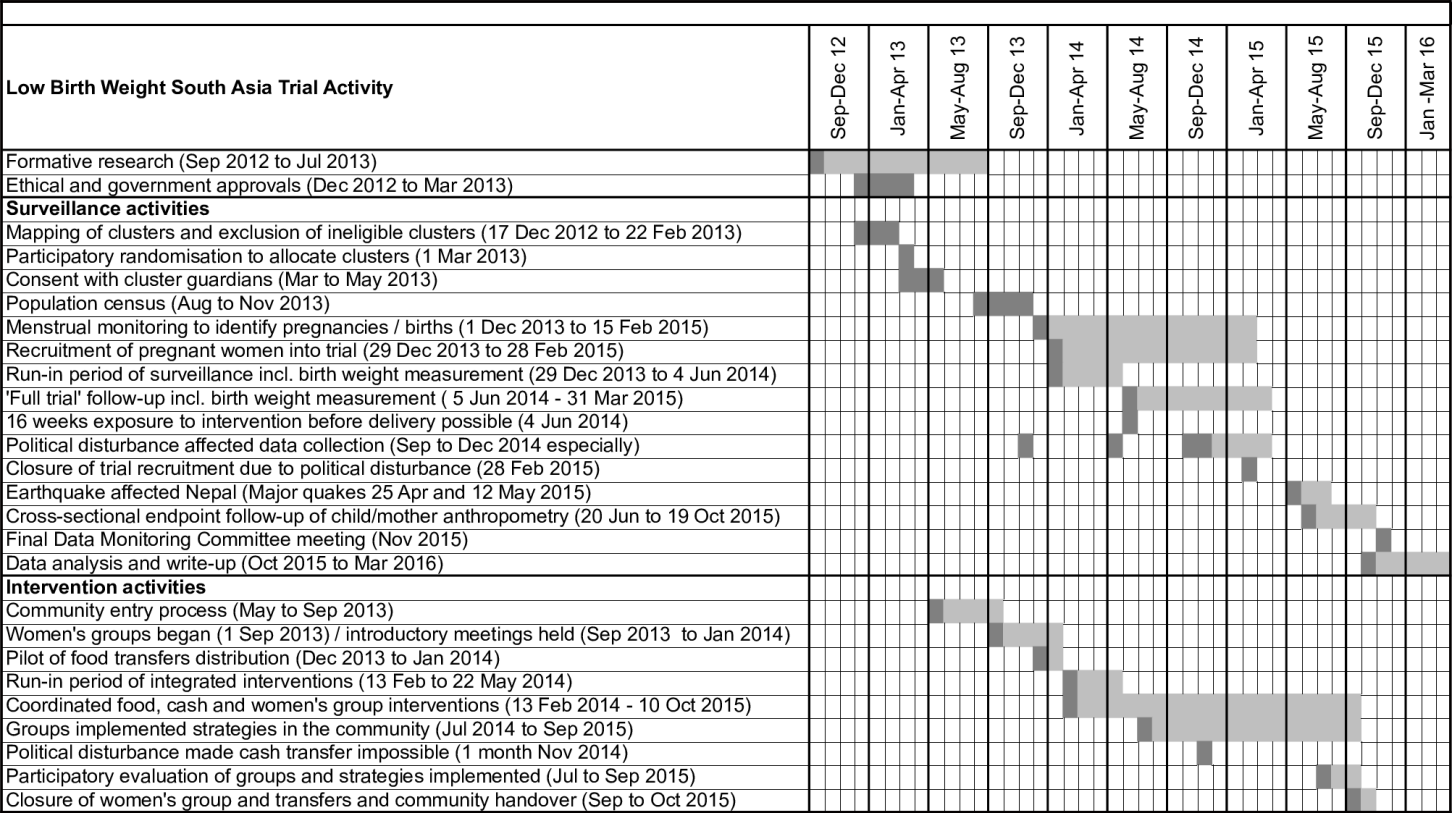
Trial timeline.

The study timeline describes the trial periods and activities (Fig 2).

Table 1 shows baseline characteristics of women eligible for analysis in the four study arms. There were no substantial differences between arms, except in the proportion of women whose husbands were working overseas, which was higher in control (31%) than in intervention arms (22–26%). Many women were aged 20–24 (38.8%) or were adolescent (37.4% were 15–19 and 0.5% 12 to 14 years), few were between 30 and 46 years (5.5%) and around one-third were primigravid. Most had no schooling (64%) and only 15% had any secondary education. Disadvantaged Dalit castes and Muslims accounted for one-third and ‘middle’ Terai caste groups accounted for 43%. Yadav or Brahmin (least poor) castes were more prevalent in the PLA plus cash arm (28%) than in the control arm (19%). Most households (74%) had no (or shared) toilet and used biomass fuels for cooking (95%).

**Table 1:** Baseline characteristics of trial participants eligible in weight-for-age z-score analysis.

	*n*	0. Control *n*, %		1. PLA only *n*, %		2. PLA + Cash *n*, %		3. PLA + Food *n*, %		Total *n*, %	
**Study arm**	10936	2426		2448		3065		2997		10936	
**Randomisation stratum**	10936										
small, inaccessible population		488	20%	561	23%	553	18%	614	20%	2216	20%
small, accessible population		549	23%	546	22%	649	21%	683	23%	2427	22%
large, inaccessible population		733	30%	648	26%	1016	33%	832	28%	3229	30%
large, accessible population		656	27%	693	28%	847	28%	868	29%	3064	28%
**Previous pregnancies**	10718										
0		792	33%	768	32%	951	32%	894	31%	3405	32%
1		641	27%	687	28%	835	28%	851	29%	3014	28%
2		485	20%	523	22%	639	21%	591	20%	2238	21%
3		281	12%	277	11%	314	10%	334	11%	1206	11%
4 or more		201	8%	157	7%	262	9%	235	8%	855	8%
**Age of pregnant woman**	10936										
12–19 y		843	35%	900	37%	1241	40%	1176	39%	4160	38%
20–24 y		992	41%	946	39%	1148	37%	1158	39%	4244	39%
25–29 y		431	18%	471	19%	513	17%	515	17%	1930	18%
30–46 y		160	7%	131	5%	163	5%	148	5%	602	6%
**Maternal education**	10715										
Never went to school		1603	67%	1475	61%	1873	62%	1904	66%	6855	64%
Primary to lower secondary		469	20%	539	22%	649	22%	550	19%	2207	21%
Secondary or higher		327	14%	397	16%	479	16%	450	15%	1653	15%
**Wealth quintile**	10622										
1 (poorest)		462	19%	456	19%	507	17%	523	18%	1948	18%
2		446	19%	508	21%	592	20%	612	21%	2158	20%
3		532	22%	461	19%	594	20%	589	21%	2176	20%
4		475	20%	451	19%	627	21%	629	22%	2182	21%
5 (least poor)		481	20%	519	22%	644	22%	514	18%	2158	20%
**Multi-dimensional poverty index**	10127										
Poorest		835	36%	782	34%	866	31%	885	33%	3368	33%
Middle		730	32%	711	31%	957	34%	929	35%	3327	33%
Least poor		750	32%	821	35%	989	35%	872	32%	3432	34%
**Husband working overseas**	9461										
No		1529	69%	1679	76%	2032	78%	1798	74%	7038	74%
Yes		694	31%	524	24%	589	22%	616	26%	2423	26%
**Caste (grouped by disadvantage)**	10936										
Dalit/Muslim- disadvantaged		901	37%	803	33%	992	32%	1057	35%	3753	34%
Janjati/Other Terai castes—mid		1076	44%	1116	46%	1229	40%	1281	43%	4702	43%
Yadav/Brahmin—least		449	19%	529	22%	844	28%	659	22%	2481	23%
**Toilet ownership**	10714										
Owns/ does not share a toilet		582	24%	669	28%	830	28%	736	25%	2817	26%
Deprived of / shares a toilet		1817	76%	1741	72%	2171	72%	2168	75%	7897	74%
**Main fuel used for cooking**	10714										
Biomass (wood, dung, crop residues)		2268	95%	2251	93%	2832	94%	2787	96%	10138	95%
Gas or other non-biomass		131	5%	159	7%	169	6%	117	4%	576	5%

Outcome measures were available in all clusters. Capture rates for data and outcomes are described in S2 Table. Birthweight was analysed for 2087 children measured within 72 hours (control: n = 464; PLA alone n = 488; PLA plus cash n = 509; PLA plus food n = 626) and WAZ for 9242 children aged 0–16 months, average age 9 months (control: n = 2091; PLA alone n = 2095; PLA plus cash n = 2549; PLA plus food n = 2507). Capture rates for birthweight were low during the main surveillance period due to unexpectedly high numbers of women enrolling, especially in transfer arms, and conflict affecting field team performance. Capture rates were balanced across arms: 22% of potential birthweights (range 19–24% per arm) and 84% of potential WAZ (range 83–86%).

PLA and food or cash transfer interventions were implemented via 539 groups in all 60 intervention clusters as planned. Coverage of PLA groups and group activities amongst women eligible for WAZ analyses is summarised in Table 2. Around 20 women attended each PLA group, with a higher proportion of pregnant women attending (usually accompanied by a female relative) in transfer arms. Attendance at PLA groups was high amongst enrolled women, especially in food and cash transfer areas where 99% of cash and 97% of food arm families had heard of groups and 98% of cash and 96% of food families had a member who attended. These proportions were significantly higher (p<0.0001) than those in the PLA only arm where 65% had heard of groups and 55% had attended or had a household member who attended, and 49% of pregnant women had themselves attended. Women in the PLA only arm attended only twice on average during pregnancy, compared with four times in PLA plus transfer arms. This may be associated with differential gestational age at enrolment. Average gestational age in months at enrolment was higher in PLA plus cash (mean 3.7, SD *1*.*4*) and PLA plus food (3.7, *1*.*5*) arms than in control (5.4, *2*.*1*) or PLA only (5.2, *2*.*1*) arms. This means that women in food or cash arms had on average 1.5 months longer to be exposed to groups than in the PLA only arm.

**Table 2:** Intervention exposure across study arms in cases eligible for weight-for-age z-score analysis.

	***n***	**0. Control**		**1. PLA only**		**2. PLA + Cash**		**3. PLA + Food**	
**Exposure to women's groups (*n)***	*9662*	*2189*	* *	*2181*	* *	*2680*	* *	*2612*	* *
Heard of women's groups (yes/no)		111	5%	1414	65%	2646	99%	2529	97%
*OR relative to PLA only (95% CI)*				*1*	(ref)	*47*.*9*	*(25*.*2*, *91*.*0)*	*24*.*9*	*(13*.*6*, *45*.*5)*
p**							*<0*.*0001*		*<0*.*0001*
Attended women's groups (yes/no)		28	1%	1207	55%	2626	98%	2514	96%
*OR relative to PLA only (95% CI)*				*1*	(ref)	*52*.*7*	*(28*.*5*, *97*.*4)*	*32*.*5*	*(18*.*0*, *58*.*7)*
p**							*<0*.*0001*		*<0*.*0001*
Times pregnant woman attended group*									
0 women's groups attended		2167	99%	1128	52%	117	4%	196	8%
1–4 women's groups attended		21	1%	739	34%	1069	40%	1062	41%
5 or more women's groups attended		1	0%	314	14%	1494	56%	1354	52%
**Times pregnant woman attended group** (mean *sd*)		0.02	*0*.*2*	1.99	*3*.*26*	4.71	*2*.*35*	4.44	*2*.*47*
PLA group* **attended *(n*, *%)***	*9507*	*2146*	* *	*2157*	* *	*2633*	* *	*2571*	* *
Pregnant woman		22	1%	1059	49%	2607	97%	2448	94%
Mother-in-law		2	0%	365	17%	1041	39%	1039	40%
Husband		0	0%	3	0%	22	1%	16	1%
Other family member		0	0%	68	3%	236	9%	210	8%
**Exposure to women's group strategy (*n*, *%)***	*9507*	*2146*	* *	*2157*	* *	*2633*	* *	*2571*	* *
Nutrition mobiliser or Female Community Health Volunteer showing picture cards		25	1%	980	45%	2427	92%	2270	88%
Home visit from nutrition mobiliser		11	1%	359	17%	983	37%	915	36%
Group members sharing group messages		2	0%	220	10%	677	26%	652	25%
Screened video on pregnancy danger signs		0	0%	8	0%	118	4%	116	5%
Nutrition rally		0	0%	2	0%	84	3%	63	2%
None of the above		2114	99%	1072	50%	121	5%	233	9%
**Exposure to extra meetings run as a group strategy *(n*, *%)***	*9507*	*2146*	* *	*2157*	* *	*2633*	* *	*2571*	* *
Pregnant women		15	1%	673	31%	1343	51%	1453	57%
Mothers-in-law		3	0%	195	9%	390	15%	419	16%
Adolescents		4	0%	100	5%	145	6%	103	4%
Sub-cluster (hamlet)		5	0%	36	2%	46	2%	153	6%
Men		2	0%	7	0%	9	0%	17	1%
Not exposed to extra groups		2119	99%	1443	67%	1252	48%	962	37%
**Exposure to social transfers**	***n***	**0. Control**		**1. PLA only**		**2. PLA + Cash**		**3. PLA + Food**	
**Super cereal food transfers received *(n*, *%)***	2574								
0–3 food transfers								546	21%
4–5 food transfers								1193	46%
6–9 food transfers^1^								835	32%
**Times pregnant woman received 10 kg Super Cereal (**mean *sd*)								4.66	*1*.*60*
**Cash transfers received *(n*, *%)***	2633								
0–3 cash transfers						605	23%		
4–5 cash transfers						1227	47%		
6–9 cash transfers^1^						801	30%		
**Times pregnant woman received NPR 750 cash transfer** (mean *sd*)						4.57	*1*.*60*		

* PLA group organized by MIRA (Mother and Infant Research Activities) implementing NGO

** comparing PLA plus cash and PLA plus food (arms 2 and 3) with PLA only (arm 1)

^1^ a small number reported receiving more than the allotted 7 transfers. This amounted to 13 cases (0.4%) in the food arm and 18 cases (0.6%) in the cash arm.

Home visits reached 17% of women in the PLA arm and 36–37% in PLA plus transfer arms. Most home visits were to invite women to attend the group or deliver transfers to women unable to attend, but some were delivered as a group strategy. Coverage of strategies implemented by PLA groups to raise community awareness about nutrition and low birth weight was higher in PLA plus transfer arms (>91%) than in the PLA only arm (50%). Exposure to community strategies was highest for viewing picture cards and attending extra meetings (targeted especially for pregnant women or mothers-in-law) and lowest for rallies and video showing. Few husbands or other men attended the groups.

In total 28,527 cash transfers were delivered to 6,651 pregnant women and 25,679 food transfers were delivered to 6,836 pregnant women. For trial participants (Table 2), 32% of women in food and 30% in cash arms received 6 or more transfers over pregnancy, 46% in food and 47% in cash arms received 4–5 transfers, and 21% in food and 23% in cash received 0–3 transfers. In a qualitative study in the PLA plus cash arm, women reported spending their cash on milk and fruit in particular [40]. In the PLA plus food arm, two-thirds of women used the Super Cereal in a sweet snack called *haluwa*, prepared by adding oil or *ghee*, sugar, and water or milk. Around one-third prepared it as *roti* (flatbread), made with water and very little or no oil. In a subsample of 768 interviewed in late pregnancy, women ate Super Cereal 1·4 times per day and 5 days per week on average, and around 43% reported sharing it with other family members. The food was thought of as ‘medicine’ for the woman and her baby. Contamination of control areas was minimal. Only 5% of control group respondents had heard of women’s groups facilitated by the implementing NGO and 1% had attended groups or observed group strategies that involved community awareness-raising.

S3 Table provides comparisons of characteristics of women whose infants’ birthweights were and were not captured. Compared with those whose infants were not measured, mothers of infants whose weights were captured were significantly older, had more children, were more likely to be Hindu than Muslim, and were less likely to have primary or secondary education. Many younger, first time mothers, who were more likely than older women to have some education, were not at the home where they had enrolled in the study at the time of the birth. This may be explained by the custom of younger pregnant women and girls (especially Muslims) with no or few previous children returning to their parental home for delivery, while mothers with two or more children stay with their marital families.

Infant anthropometric outcomes are presented in Table 3. Relative to the control arm (mean 2756 g, n = 464) birthweight measured within 72 hours (the birthweight primary outcome) appeared to be incrementally higher in PLA (28·9 g; *95% CI -37·7*, *95·4; n = 488*), PLA plus cash (50·5 g; *95% CI -15·0*, *116·1; n = 509*), and PLA plus food (78·0 g; *95% CI 13·9*, *142·0; n = 629*) arms, but the only significant difference was between control and PLA plus food. The difference of 49 g (*95% CI -13·5*, *111·8)* between PLA only and PLA plus food was not significant. LBW prevalence was lower in the PLA plus food arm (19.1% OR 0.74, *95% CI 0*.*51*,*1*.*08*) than in the control arm (22.5%), representing a non-significant 16% decrease associated with the intervention.

**Table 3:** Effect of interventions on birth weight and infant anthropometry in first 42 days.

Outcome	Parameter	0. Control		1. PLA only			2. PLA + Cash			3. PLA + Food		
	Total eligible (all arms = 9786) (N)	2165		2198			2716			2707		
Age category at time of weight measurement 0–42 days	All infant weights 0 to 42 days (N)	922		836			956			1045		
	within 72 hours (n *%*)	476	52%	497		59%	525		55%	649		62%
	72 hours to 10 days (n %)	139	15%	110		13%	141		15%	150		14%
	10–42 days (n %)	307	33%	229		27%	290		30%	246		24%
**Primary outcome: Birth weight in grams within 72 hours** *N used in analysis = 2087*	Mean	2755.7		**2780.7**			**2802.1**			**2830.3**		
	SD	395.6		*434*.*3*			*434*.*5*			*432*.*5*		
	n	476		497			525			649		
	Capture rate (%)	22.0%		22.6%			19.3%			24.0%		
	Adjusted effect measure grams increase in weight^1^^,^^2^ *(95% CI)*	0 (ref)		27.6			50.2			**78.1**		
				*-37*.*4*	,	*92*.*7*	*-13*.*9*	,	*114*.*3*	***15*.*6***	,	***140*.*5***
	p			0.4051			0.1310			**0.0143**		
	n in analysis	464		488			509			626		
	Adjusted birth weight effect measure relative to PLA only^1^^,^^2^ (*95% CI*)			0 (ref)						50.4		
										*-10*.*8*	,	*111*.*6*
	p									0.1060		
	Low Birth Weight below 2500g	22.5%		**20.9%**			**21.9%**			**19.1%**		
	Adjusted Odds Ratio for LBW relative to control^1^^,^^2^ *(95% CI)* ^&^	0 (ref)		0.85			0.90			0.74		
				*0*.*59*	,	*1*.*22*	*0*.*62*	,	*1*.*31*	*0*.*50*	,	*1*.*12*
	p			0.3795			0.5852			0.1516		
Weight in grams within 10 days* †^&^ *N used in analysis = 2606 (including the 2087 used for birthweight within 72 hours)*	Mean	2770.2		**2792.4**			**2833.9**			**2839.0**		
	SD	403.7		*439*.*2*			*434*.*8*			*432*.*0*		
	n	615		607			666			799		
	Adjusted effect measure in grams weight increase^1^^,^^2^ *(95% CI)*	0 (ref)		36.1			**68.8**			**72.4**		
				*-30*.*8*	,	*103*.*1*	***3*.*2***	,	***134*.*4***	***7*.*5***	,	***137*.*2***
	*p*			0.2903			**0.0397**			**0.0288**		
	Adjusted effect measure relative to PLA only^1^^,^^2^ (*95% CI*)			0 (ref)			32.7			36.2		
							*-32*.*4*	,	*97*.*7*	*-28*.*1*	,	*100*.*6*
	p						0.3250			0.2700		
Weight-for-age Z-score within 42 days**† ^&^ *N used in analysis = 3624 (including the 2606 used for birthweight within 10 days)*	Mean	-1.29		-1.22			-1.18			-1.11		
	SD	1.02		*1*.*07*			*1*.*05*			*1*.*02*		
	n	922		836			956			1045		
	Adjusted effect measure WAZ within 42 days^1^^,^^2^ *(95% CI)*	0 (ref)		0.065			0.104			**0.154**		
				*-0*.*068*	,	*0*.*198*	*-0*.*025*	,	*0*.*233*	***0*.*025***	,	***0*.*283***
	p			0.3358			0.1129			**0.0191**		
	Adjusted effect measure relative to PLA only^1^^,^^2^ (9*5% CI*)			0 (ref)						0.089		
										*-0*.*042*	,	*0*.*220*
	p									0.1850		
Under weight <-2 WAZ**	*Freq*	191		164			191			180		
	%	*20*.*7*		*19*.*6*			*20*.*0*			*17*.*2*		
Length in cm within 10 days*†^&^ *N used in analysis = 2576*	Mean	47.91		47.71			48.06			47.97		
	*SD*	*2*.*24*		*2*.*20*			*2*.*19*			*2*.*03*		
	n	610		606			657			784		
	Adjusted effect measure length within 10 days^1^^,^^2^ *(95% CI)*	0 (ref)		-0.440			0.070			-0.076		
				*-1*.*058*	,	*0*.*177*	*-0*.*542*	,	*0*.*683*	*-0*.*693*	,	*0*.*540*
	p			0.1624			0.8217			0.8079		
Length-for-age Z-score within 10 days*$ ^&^ *N used in analysis = 2572*	Mean	-1.08		-1.14			-0.98			-1.03		
	*SD*	1.16		*1*.*05*			*1*.*13*			*1*.*06*		
	n	610		606			657			784		
Stunted <-2 LAZ within 10 days*	*Freq*	115		106			97			122		
	%	*18*.*9*		*17*.*5*			*14*.*8*			*15*.*6*		
Length-for-age Z-score within 42 days**$	Mean	-1.10		-1.21			-1.03			-1.05		
	*SD*	1.24		*1*.*13*			*1*.*16*			*1*.*12*		
	n	915		831			939			1022		
Stunted <-2 LAZ within 42 days**	*Freq*	189		179			163			182		
	%	*20*.*6*		*21*.*4*			*17*.*4*			*17*.*8*		
Weight-for-length Z-score within 42 days**$	Mean	-1.289		-1.224			-1.177			-1.114		
	SD	1.023		*1*.*073*			*1*.*047*			*1*.*016*		
	n	922		836			956			1045		
Head circumference in cm within 10 days* ^&^ *N used in analysis = 2588*	Mean	33.22		33.48			33.48			33.48		
	*SD*	*1*.*46*		*1*.*39*			*1*.*53*			*1*.*32*		
	n	605		607			660			794		
	Adjusted effect measure head circumference within 10 days^1^^,^^2^ *(95% CI)*	0 (ref)		0.174			0.161			0.199		
				*-0*.*173*	,	*0*.*521*	*-0*.*183*	,	*0*.*504*	*-0*.*146*	,	*0*.*544*
	p			0.3253			0.3593			0.2575		

^1^ excluding multiple births, infants born to dead mothers, infants who died in first 72 hours before birth weight, infants with congenital abnormality.

^2^ adjusted for child age, sex, randomisation stratum, adolescence, gravida, caste, wealth score, maternal education, and husband's labour migration.

* includes measures taken within 72 hours.

** includes measures taken within 72 hours, between 72 hours and 10 days and between 10 and 42 days

† Additional outcome added in analysis plan.

$ Secondary outcome not selected for analysis in the analysis plan.

^&^ outcomes analysed at request of reviewers despite <40% response rates.

When birthweights measured within 10 days were analysed (including those measured within 72 hours) as a secondary outcome, differences from control were not significant for PLA alone (36.1 g; *95% CI -30*.*8*, *103*.*1*) but were significant for PLA plus cash (68.8 g; *95% CI 3·2*, *134·4*) and PLA plus food (72.4 g; *95% CI 7·5*, *137·2*). Differences between these and the PLA only arm (32–36 g) were not significant. The effect of food arm on infant size was sustained when weight-for-age (WAZ) scores from 0 to 42 days were analysed. Infants in the food arm had mean WAZ scores 0.154 (*95% CI 0*.*025*, *0*.*283*) greater than control but there was no difference between control and other arms PLA only (0.065 g; *95% CI -0*.*068*, *0*.*198*) and PLA plus cash (0.154g; *95% CI -0*.*025*, *0*.*233*). Amongst these 57% of weights were measured within 72 hours, 14% from 72 hours to 10 days, and 29% from 10 to 42 days.

No significant differences were seen in infant head circumference or infant length within 10 days. Length-for-age z-score (LAZ) measures within 10 days suggest that stunting (<-2 length for age z score) prevalence was lower than control in PLA plus cash and PLA plus food arms (18.9%, 14.8%, 15.6% respectively) but formal analyses were not undertaken following the analysis plan.

Table 4 shows maternal eating behaviour and maternal and newborn morbidity by study arm. Although response rates were lower than the 40% cut-off in our analysis plan, at the request of reviewers of this article, we tentatively analysed a small number outcomes only as shown. The findings suggest that higher proportions of pregnant women in intervention arms had an adequate number of food groups in the previous 24 hours compared with control, especially in the PLA plus cash arm (54% versus 39% in control), but this association did not reach significance. The possible difference may be attributed to increased consumption of dairy foods (64% versus 53% in control). Women in the cash arm consumed a significantly higher number of meals per day during pregnancy. Mother’s weight in late pregnancy was somewhat higher in all intervention arms compared with control.

**Table 4:** Outcomes for maternal anthropometry, diet in pregnancy, maternal morbidity in pregnancy and post-partum and neonatal morbidity, for which formal analyses are not conducted due to low response.

Outcome	N	0. Control		1. PLA only		2. PLA + Cash		3. PLA + Food	
**Maternal eating behaviour in pregnancy**	** **	** **	** **			** **	** **		** **
Maternal weight in late pregnancy (kg: mean sd) ^†^^&^	1799	50.7	*5*.*8*	50.9	*6*.*5*	50.9	*6*.*9*	51.3	*6*.*5*
Coeff. (95% CI)		0 (ref)		0.379	(-1.038, 1.797)	0.503	(-0.799, 1.805)	0.515	(-0.837, 1.868)
p					0.5999		0.4490		0.4552
Maternal weight gain (g) per week (mean sd)	519	312.4	*254*.*8*	333.9	*336*.*5*	358.2	*272*.*4*	368.2	*234*.*2*
Eating pattern in late or early pregnancy	3064								
*Ate less than pre-pregnancy*		261	39%	166	28%	337	34%	375	47%
*Ate same as pre-pregnancy*		336	50%	323	55%	421	42%	314	39%
*Ate more than pre-pregnancy*		79	12%	98	17%	241	24%	113	14%
Dietary diversity score (of 10) (mean sd) ^&^	2913	4.2	*1*.*5*	4.4	*1*.*5*	**4.7**	*1*.*6*	4.4	*1*.*4*
Coeff. (95% CI)		0 (ref)		0.196	(-0.263, 0.655)	0.552	**(0.115, 0.989)**	0.146	(-0.301, 0.593)
p					0.4020		**0.0133**		0.5217
Adequate dietary diversity in last 24 h	2913	254	39%	247	43%	509	**54%**	343	46%
Ate dairy in last 24 h	3064	359	53%	331	56%	641	**64%**	433	54%
Ate vitamin-A/ iron rich fruits, vegetables in last 24 h	3064	256	38%	238	41%	402	40%	320	40%
Ate flesh foods in last 24 h	3064	196	29%	192	33%	317	32%	236	29%
Eating occasions per day (mean sd) ^&^	3064	3.33	*0*.*99*	3.43	*0*.*94*	**3.58**	*0*.*97*	3.35	*0*.*82*
Coeff. (95% CI)		0 (ref)		0.080	(-0.146, 0.306)	0.292	**(0.079, 0.504)**	0.026	(-0.191, 0.244)
p					0.4886		**0.0071**		0.8121
Avoided foods in early or late pregnancy	833	77	55%	87	58%	169	54%	102	44%
Fasted in pregnancy	1695	373	100%	321	99%	537	100%	457	100%
**Maternal morbidity**	** **	** **	** **			** **	** **		** **
Excessive vaginal bleeding during labour	2224	17	2%	21	4%	23	5%	18	4%
Excessive vaginal bleeding after delivery	2224	78	11%	75	13%	35	7%	25	6%
Swollen face in pregnancy or postpartum	2224	136	19%	76	13%	86	17%	27	7%
Convulsions in pregnancy or postpartum	2224	83	11%	75	13%	31	6%	21	5%
High Blood pressure in pregnancy or postpartum	2224	58	8%	19	3%	21	4%	6	1%
Retained placenta	2224	72	10%	25	4%	20	4%	11	3%
Pica, spoon nail, paleness or stomatitis in late preg’	1801	48	11%	62	18%	79	14%	88	20%
Shortness of breath doing everyday tasks in pregnancy	1801	123	29%	84	24%	129	23%	130	29%
Tiredness, lethargy, weakness in pregnancy	1801	241	56%	178	50%	236	41%	196	44%
Severe vomiting 4 +times per day with weight loss	1760	29	7%	29	8%	22	4%	9	2%
Night blindness in pregnancy	1801	13	3%	17	5%	13	2%	15	3%
**Neonatal morbidity (0–28 d)**									
1 or more of 7 danger signs indicated with ‘*’ below^&^	2211	275	*38%*	223	*38%*	221	*45%*	160	*40%*
Coeff. (95% CI)		0 (ref)		-0.010	(-0.137, 0.118)	0.058	(-0.071, 0.187)	-0.015	(-0.154, 0. 124)
p					0.8832		0.3794		0.8333
Acute respiratory infection symptom(s) + fever	2211	96	13%	100	17%	60	12%	58	14%
Cough for 4 or more d	2211	193	26%	157	27%	179	37%	104	26%
Noisy breathing, grunting or wheezing	2187	154	21%	106	18%	90	18%	68	17%
Fast breathing *	2187	109	15%	97	17%	91	19%	52	13%
Intercostal recession *	2187	52	7%	61	11%	54	11%	36	9%
Fever *	2211	146	20%	136	23%	114	23%	106	26%
Umbilical redness or discharge	2211	67	9%	86	15%	59	12%	24	6%
Diarrhoea	2211	63	9%	55	9%	74	15%	45	11%
Became cold to the touch *	2187	46	6%	62	11%	38	8%	29	7%
Stopped sucking 1–28 d after birth *	2191	46	6%	45	8%	46	9%	26	7%
Any eye infection	2211	38	5%	39	7%	33	7%	19	5%
Baby unresponsive *	2193	27	4%	19	3%	13	3%	7	2%
Weak or absent cry	2211	24	3%	37	6%	19	4%	24	6%
>10 skin pustules or 1 big pustule	2211	20	3%	37	6%	25	5%	24	6%
Jaundice with yellow eyes or palms	2187	19	3%	36	6%	28	6%	27	7%
Convulsions *	2187	7	1%	13	2%	9	2%	3	1%

† secondary outcome added in analysis plan due to low response rate for weight gain

^&^ outcomes analysed at request of reviewers despite <40% response rates

Differences in neonatal morbidity using one or more of seven danger signs for neonatal sepsis [41] were tested but showed no clear differences. Across all arms, mothers of 38–45% of newborns reported one or more of these danger signs and 12–17% recalled signs of acute respiratory infection with fever, which illustrates the high levels of morbidity in the population.

S4 Table shows birth outcomes and maternal deaths for the trial. Anthropometric outcomes collected at endpoint for child and mother are presented in Table 5. Table 6 shows breastfeeding, infant and young child feeding, morbidity, gestational age and institutional delivery outcomes. Analysis of the endpoint WAZ primary outcome indicated little difference between arms. Relative to the control arm (n = 2091) the mean adjusted differences in WAZ after imputation in the PLA alone, PLA plus cash and PLA plus food arms were -0·026 (95% CI -0·117, 0·065; n = 2095), -0·045 (95% CI -0·133, 0·044; n = 2549) and -0·033 (95% CI -0·121, 0·056; n = 2507), respectively.

**Table 5:** Analysis of anthropometric outcomes captured at endpoint by study arm.

Outcome	N	Parameter	0. Control			1. PLA only			2. PLA + Cash			3. PLA + Food		
Eligible cases for inclusion in analysis	10,936	n			2426			2448			3065			2997
Child age in months	9496	Mean, *sd*, n	8.59	*3*.*35*	2144	8.98	*3*.*39*	2153	9.29	*3*.*43*	2631	8.71	*3*.*36*	2568
**Age Category**	** **	** **	** **		** **	** **		** **	** **		** **	** **		** **
0–90 days (0-3m)	** **	n, %	123	*5*.*7*		77	*3*.*6*		99	*3*.*8*		134	*5*.*2*	** **
91–180 days (4-6m)	** **	n, %	377	*17*.*6*		387	*18*.*0*		405	*15*.*4*		455	*17*.*7*	** **
181–270 days (7-9m)	** **	n, %	595	*27*.*8*		554	*25*.*7*		605	*23*.*0*		673	*26*.*2*	** **
271–365 days (10-12m)	** **	n, %	680	*31*.*7*		669	*31*.*1*		863	*32*.*8*		819	*31*.*9*	** **
12–18 months	** **	n, %	369	*17*.*2*		466	*21*.*6*		657	*25*.*0*		486	*18*.*9*	** **
**Female**	** **	n, %, N	1063	47.2	2250	1036	46.2	2244	1264	46.4	2725	1260	47.5	2651
**Child weight kg**	9276	Mean, *sd*, n	6.89	*1*.*22*	2095	6.98	1.23	2098	6.99	1.23	2564	6.88	1.23	2519
WAZ capture rate					86%			86%			83%			84%
Underweight <-2 WAZ*		n, %	741	*35*.*44*	2091	744	*35*.*5*	2095	972	*38*.*1*	2549	917	*36*.*6*	2507
**Primary outcome: Child weight-for-age Z-score**	9242	Mean, *sd*, n	-1.60	*1*.*14*		-1.63	*1*.*11*		-1.68	*1*.*10*		-1.64	*1*.*13*	
	8024	Coeff. (95% CI)	0 (ref)			-0.044	*(-0*.*129*,	*0*.*040)*	-0.056	*(-0*.*139*,	*0*.*028)*	-0.064	*(-0*,*148*,	*0*.*019)*
						p =	0.3038		p =	0.1901		p =	0.1313	
	9242	Coeff. *(95% CI) + imputation*	0 (ref)			-0.026	*(-0*.*117*,	*0*.*064)*	-0.045	*(-0*.*134*,	*0*.*044)*	-0.033	*(-0*.*122*,	*0*.*055)*
						p =	0.5690		p =	0.3190		p =	0.4630	
**Child length, cm**	9438	Mean, *sd*, n	66.89	*5*.*28*	2139	67.39	5.29	2133	67.51	5.22	2616	66.86	5.28	2550
Stunted <-2 LAZ**		n, %, N	617	29.1	2122	622	29.3	2122	796	30.7	2595	756	29.9	2525
**Child length-for-age Z-score**		Mean, *sd*, N	-1.33	*1*.*36*		-1.34	*1*.*32*		-1.43	*1*.*25*		-1.38	*1*.*30*	
	8119	Coeff. (95% CI)	0 (ref)			-0.036	*(-0*.*156*,	*0*.*084)*	-0.068	*(-0*.*186*,	*0*.*050)*	-0.101	*(-0*.*220*,	*0*.*018)*
						p =	0.5565		p =	0.2588		p =	0.0956	
	9320	Coeff. *(95% CI) + imputation*	0 (ref)			-0.004	*(-0*.*125*,	*0*.*117)*	-0.054	*(-0*.*173*,	*0*.*066)*	-0.050	*(-0*.*170*,	*0*.*069)*
						p =	0.9490		p =	0.3740		p =	0.4100	
Wasted <-2 WLZ***		n, %	362	*17*.*4*	2086	277	*18*.*0*	2090	508	*19*.*9*	2553	461	*18*.*5*	2498
**Child weight-for-length Z-score**		Mean, *sd*, n	-1.09	*1*.*05*		-1.12	*1*.*02*		-1.14	*1*.*05*		-1.11	*1*.*03*	
	8024	Coeff. (95% CI)	0 (ref)			-0.029	*(-0*.*115*,	*0*.*056)*	-0.011	*(-0*.*096*,	*0*.*073)*	-0.026	*(-0*.*111*,	*0*.*059)*
						p =	0.5030		p =	0.7899		p =	0.5436	
**Child head circumference, cm**		Mean, *sd*, n	41.91	*2*.*24*	2131	42.13	*2*.*26*	2135	42.13	*2*.*29*	2612	41.97	*2*.*31*	2539
	8166	Coeff. (95% CI)	0 (ref)			0.014	*(-0*.*136*,	*0*.*163)*	-0.107	*(-0*.*254*,	*0*.*040)*	-0.061	*(-0*.*209*,	*0*.*087)*
						p =	0.8578		p =	0.1550		p =	0.4173	
**Mother's BMI at nutrition clinic, kg/m**^**2**^		Mean, *sd*, n	19.71	2.66	2086	19.83	2.62	2106	19.80	2.54	2580	19.74	2.54	2565
	7943	Coeff. (95% CI)	0 (ref)			0.141	*(-0*.*044*,	*0*.*325)*	0.172	*(-0*.*008*,	*0*.*353)*	0.063	*(-0*.*120*,	*0*.*246)*
						p =	0.1355		p =	0.0604		p =	0.4993	
**Mother's MUAC at nutrition clinic, cm**		Mean, *sd*, n	23.68	2.41	2098	23.70	2.34	2106	23.76	2.36	2582	23.64	2.33	2503
	7991	Coeff. (95% CI)	0 (ref)			0.009	*(-0*.*195*,	*0*.*213)*	0.133	*(-0*.*067*,	*0*.*334)*	-0.041	*(-0*.*244*,	*0*.*161)*
						p =	0.9304		p =	0.1929		p =	0.6880	

Z scores calculated using WHO 2006 growth standard

* WAZ: weight for age z-score

** LAZ: length for age z-score

*** LHZ: weight for age z-score

**Table 6:** Analysis of child feeding, morbidity and delivery-related outcomes captured at endpoint by study arm.

Outcome	N	Parameter	0. Control			1. PLA only			2. PLA + Cash			3. PLA + Food		
**Time of initiation of breastfeeding**^**1**^	3313	N			995			833			738			747
after 24 hours		n (%)	173	*17*.*4*		146	*17*.*5*		141	*19*.*1*		73	*9*.*8*	
1–24 hours		n (%)	473	*47*.*5*		370	*44*.*4*		305	*41*.*3*		282	*37*.*8*	
in 1st hour		n (%)	349	*35*.*1*		317	*38*.*1*		292	*39*.*6*		392	***52*.*5***	
	1074 (79 clusters only)	OR *(95% CI)*	0 (ref)			0.98	*(0*.*70*,	*1*.*37)*	1.39	*(0*.*99*,	*1*.*94)*	1.53	*(1*.*10*,	*2*.*14)*
						p =	0.9010		p =	0.0570		p =	**0.0110**	
**Exclusive breastfeeding**^**2**^		n, %, N	209	*43*.*8*	477	215	*47*.*0*	457	240	*50*.*4*	476	241	*44*.*3*	544
	1808	OR *(95% CI)*	0 (ref)			1.41	*(0*.*88*,	*2*.*27)*	1.54	*(0*.*97*,	*2*.*45)*	1.11	*(0*.*70*,	*1*.*75)*
						p =	0.1490		p =	0.0664		p =	0.6535	** **
**Discarded colostrum**		n, %, N	550	*27*.*49*	2001	494	*23*.*67*	2087	604	*23*.*91*	2526	555	***21*.*78***	2548
	7915	OR *(95% CI)*	0 (ref)			0.87	*(0*.*66*,	*1*.*14)*	0.81	*(0*.*61*,	*1*.*06)*	0.71	*(0*.*54*,	*0*.*93)*
						p =	0.3213		p =	0.1166		p =	**0.0137**	** **
**Continued breastfeeding beyond 6 months**^**3**^		n, %, N	1585	*98*.*33*	1608	1608	*97*.*99*	1641	2013	*98*.*39*	2044	1883	*97*.*92*	1923
	6157	OR *(95% CI)*	0 (ref)			0.89	*(0*.*47*,	*1*.*68)*	1.15	*(0*.*60*,	*2*.*20)*	0.75	*(0*.*40*,	*1*.*38)*
						p =	0.7263		p =	0.6628		p =	0.3548	
**Child Dietary Diversity Score last 24 hours** ^**3**^		Mean, *sd*, n	1.60	1.41	1544	1.72	1.40	1630	1.75	1.44	2046	1.57	1.40	1925
	5936	Coeff. (95% CI)	0 (ref)			0.042	*(-0*.*138*,	*0*.*222)*	0.040	*(-0*.*139*,	*0*.*218)*	-0.077	*(-0*.*258*,	*0*.*104)*
						p =	0.6482		p =	0.6632		p =	0.4035	
**Child consumed green leafy or yellow vegetables / fruit last 24 hours** ^**3**^ ^$^		n, %, N	191	*12*.*4*	1544	172	*10*.*6*	1630	222	*11*.*1*	2046	214	*11*.*8*	1925
	5936	OR *(95% CI)*	0 (ref)			1.42	*(0*.*82*,	*2*.*46)*	1.35	*(0*.*78*,	*2*.*34)*	0.92	*(0*.*51*,	*1*.*67)*
						p =	0.2085		p =	0.2765		p =	0.7886	
**Child consumed flesh food last 24 hours** ^**3**^ ^$^		n, %, N	55	*2*.*74*	1544	72	*3*.*45*	1630	96	*3*.*93*	2468	65	*2*.*82*	2338
	5936	OR *(95% CI)*	0 (ref)			0.76	*(0*.*46*,	*1*.*23)*	0.77	*(0*.*47*,	*1*.*25)*	0.78	*(0*.*47*,	*1*.*28)*
						p =	0.2605		p =	0.2861		p =	0.3230	
**Child unwell last 2 weeks**		n, %, N	1172	*55*.*94*	2095	1078	*51*.*24*	2104	1340	*52*.*41*	2557	1266	*50*.*42*	2511
	8027	OR *(95% CI)*	0 (ref)			0.89	*(0*.*72*,	*1*.*10)*	0.87	*(0*.*71*,	*1*.*07)*	0.90	*(0*.*73*,	*1*.*11)*
						p =	0.2979		p =	0.1975		p =	0.3340	
**Delivered at health institution**		n, %, N	783	*34*.*78*	2251	836	*37*.*24*	2245	1045	*38*.*35*	2725	1113	***41*.*98***	2651
	8598	OR *(95% CI)*	0 (ref)			1.04	*(0*.*74*,	*1*.*48)*	1.08	*(0*.*76*,	*1*.*53)*	1.45	*(1*.*03*,	*2*.*06)*
						p =	0.8143		p =	0.6658		p =	**0.0344**	
**Child born preterm before 37 weeks using est. gestation**		n, %, N	319	*14*.*17*	2251	305	*13*.*59*	2245	355	*13*.*03*	2724	330	*12*.*45*	2651
	8594	OR *(95% CI)*	0 (ref)			0.98	*(0*.*81*,	*1*.*18)*	0.92	*(0*.*77*,	*1*.*11)*	0.90	*(0*.*74*,	*1*.*08)*
						p =	0.8124		p =	0.3823		p =	0.2485	
**Gestational age at birth** ^$^		Mean, *sd*, n	287.9	68.1	2251	288.1	65.9	2245	286.1	59.8	2724	285.5	57.6	2651
	8594	Coeff. (95% CI)	0 (ref)			-1.067	*(-6*.*172*,	*4*.*038)*	-2.681	*(-7*.*698*,	*2*.*336)*	-4.755	*(-9*.*817*,	*0*.*307)*
						p =	0.6820		p =	0.2949		p =	0.0656	

^1^ on a subsample of exclusive breast feeders with acceptable response rate only

^2^ in children up to 180 days only

^3^ in children over 180 days

^$^ secondary outcome added in analysis plan to represent key behaviours that were promoted in the PLA meetings

There was no effect of pregnancy interventions on maternal anthropometry 0–16 months after birth (BMI, underweight, MUAC) or on child anthropometry (WAZ, LAZ, WLZ, head circumference), preterm birth or gestational age at delivery, infant feeding behaviours, including continued breastfeeding beyond 6 months, child dietary diversity score, consumption of green leafy vegetables and fruits, flesh foods, or child morbidity. However, significantly more institutional deliveries (OR 1·46; 95% CI 1·03, 2·06; n = 2651) and lower rates of colostrum discarding (OR 0·71, 95% CI 0·54, 0·93; n = 2548) were observed in the PLA plus food arm compared with control (institutional delivery n = 2251; colostrum n = 2087).

Comparing average WAZ between time periods after birth (-1·11 to -1·29 z-scores within 42 days and -1·60 to -1·68 z-scores at age 9 months on average), children became more underweight between early infant anthropometry and endpoint in all study arms. Prevalence of stunting increased between 42 days (17·4%-21·4% across study arms) and 9 months on average (29·1%-30·7% across study arms).

Dose-response analyses (reported with imputation) in Table 7 show that, in the PLA only arm, attending more groups was linked to higher WAZ at 9 months age on average, whereas in the PLA plus cash arm more transfers were associated with lower WAZ. More cash transfers were also associated with lower LAZ. There was no significant dose-response effect in the food arm. S5 Table shows no association between socioeconomic status and the effect of any of the three interventions.

**Table 7:** Dose-response analyses of the effect of pregnancy intervention intensity on weight-for-age and length-for-age z-scores measured at endpoint (0 to 16 months, average age 9 months), with and without multiple imputation of covariates.

	**Weight-for-age z-score (no imputation)**			**Weight-for-age z-score with imputation of co-variates**		
**Exposures**	Coeff	*95% CI*	*Within-arm test for dose response (p)*	Coeff	*95% CI*	*Within-arm test for dose response (p)*
Control arm	0			0		
0 women’s groups attended	-0.072	*(-0*.*172*,*0*.*028)*	0.2218	-0.072	*(-0*.*167*,*0*.*024)*	0.0337
1–4 women’s groups attended	-0.054	*(-0*.*163*,*0*.*055)*		-0.009	*(-0*.*113*,*0*.*096)*	
5+ women’s groups attended	0.062	*(-0*.*081*,*0*.*206)*		0.091	*(-0*.*048*,*0*.*230)*	
0–3 cash transfers received	0.014	*(-0*.*105*,*0*.*132)*	0.0037	0.043	*(-0*.*068*,*0*.*155)*	<0.0001
4–5 cash transfers received	-0.053	*(-0*.*151*,*0*.*044)*		-0.060	*(-0*.*152*,*0*.*033)*	
6+ cash transfers received	-0.111	*(-0*.*219*,*-0*.*003)*		-0.092	*(-0*.*196*,*0*.*011)*	
0–3 food transfers received	0.000	*(-0*.*126*,*0*.*124)*	0.6478	0.035	*(-0*.*079*,*0*.*150)*	0.4510
4–5 food transfers received	-0.107	*(-0*.*205*,*-0*.*009)*		-0.073	*(-0*.*165*,*0*.*020)*	
6+ food transfers received	-0.043	*(-0*.*152*,*0*.*066)*		-0.020	*(-0*.*122*,*0*.*081)*	
	n = 8034			n = 9242		
	**Length-for-age z-score (no imputation)**			**Length-for-age z-score with imputation of co-variates**		
Control arm	0			0		
0 women’s groups attended	-0.025	*(-0*.*160*,*0*.*110)*	0.5345	-0.011	*(-0*.*142*,*0*.*120)*	0.6503
1–4 women’s groups attended	-0.069	*(-0*.*213*,*0*.*075)*		-0.017	*(-0*.*157*,*0*.*123)*	
5+ women’s groups attended	-0.002	*(-0*.*181*,*0*.*177)*		0.048	*(-0*.*129*,*0*.*223)*	
0–3 cash transfers received	0.174	*(0*.*021*,*0*.*327)*	<0.0001	0.197	*(0*.*050*,*0*.*344)*	<0.0001
4–5 cash transfers received	-0.124	*(-0*.*256*,*0*.*008)*		-0.127	*(-0*.*256*,*0*.*001)*	
6+ cash transfers received	-0.164	*(-0*.*307*,*-0*.*021)*		-0.136	*(-0*.*275*,*0*.*002)*	
0–3 food transfers received	0.013	*(-0*.*147*,*0*.*172)*	0.1580	0.103	*(-0*.*047*,*0*.*253)*	0.0490
4–5 food transfers received	-0.174	*(-0*.*307*,*-0*.*041)*		-0.136	*(-0*.*264*,*-0*.*008)*	
6+ food transfers received	-0.069	*(-0*.*212*,*0*.*075)*		-0.027	*(-0*.*164*,*0*.*111)*	
	n = 8119			n = 9320		

## Discussion

Our trial of the effects of PLA women’s groups, with and without transfers of cash or food to pregnant women, suggests an increase in birthweight in the PLA plus food arm. However, better nutritional status soon after birth did not persist into early childhood, reflected by only small differences between arms in child anthropometry at 0–16 months. No intervention effects were seen for many secondary outcomes and the number of secondary outcomes tested prevents firm conclusions, but in the PLA plus food arm the prevalence of colostrum discarding was lower and the number of institutional deliveries was higher. We conclude that adding a fortified balanced energy protein supplement to a Participatory Learning and Action behaviour change intervention is likely to increase birthweight, and may be more effective than providing a modest amount of cash with PLA or conducting PLA alone. Sustaining the improvement in birthweight over infancy may require intervention beyond pregnancy, including nutritional support to lactating women and age-appropriate complementary feeding and consideration of other environmental challenges.

The study limitations relate largely to the compromised capture of birthweights during ethnic conflict in the data collection team, and consequent lack of robustness to our birthweight findings. Food and cash distribution were delayed during monsoons due to road access problems, and cash transfers could not be delivered in October 2014 because of security concerns.

As there are strong geographical and cultural similarities, our findings are likely to be generalizable within the plains of Nepal and to other South Asian populations living in plains areas with predominant rice production in India, Bangladesh, and Pakistan. Strong cultural differences between hill and plain Nepalese communities mean that they may be less generalizable to hilly areas of Nepal with greater food insecurity or where women’s freedom to move beyond their home environment is less restricted.

The effect of Super Cereal (fortified sweetened wheat-soy blended flour) supplement on birthweight in the context of a PLA intervention, and the lack of appreciable effect of PLA alone, is consistent with several reviews that showed increases from protein-energy supplements [11–14]. In contrast with our findings, pregnancy supplementation with a World Food Programme Super Cereal-like blend of corn rather than wheat in Cambodia showed no effect on birthweight or mother’s weight gain in pregnancy, although there was a trend for higher impact among women with a lower BMI as compared to women with a higher BMI in early pregnancy, and a decrease was found in preterm delivery and anaemia [15].

Although PLA alone has been effective in reducing neonatal mortality [27], and in changing nutrition-related behaviours [25, 30, 31, 42], effects of PLA alone on birthweight were not large enough to reach significance in our study. This is consistent with the lack of effect of PLA plus home visits on birthweight found by Nair et al [25]. Whilst PLA differs from conventional nutrition education (NE) due to strategy implementation mobilising communities, the lack of impact on birthweight of PLA alone is somewhat similar to Girard’s review, which showed substantial increases in birthweight after NE with nutrition support in the form of food or micronutrient supplements, but no increase after NE alone [19]. A review of fifteen studies of nutrition counselling alone also showed varying effects [11].

The lack of sustained difference in WAZ appears to contrast with the follow-up of an antenatal multiple micronutrient supplementation trial in the same districts of Nepal [43], which showed a 205 g difference between intervention and control children at two years, having demonstrated a 77 g difference at birth [44]. The population in the multiple micronutrient study was more urbanised and generally less poor than our cohort, and the difference had disappeared by nine years of age [45]. Our endpoint data collection occurred during monsoon when levels of diarrhoeal disease and child underweight were high.

Other studies have also found that the effects of pregnancy food supplementation were inconsistent in childhood. For example, the MINIMat study in Bangladesh compared the effects of early and late pregnancy supplementation with food and micronutrients on child growth in the first 54 months. Compared with late pregnancy supplementation, early supplementation was associated with reduced stunting in boys, but not girls. However, as in our study, no effect was seen on weight-for-age or weight-for-height z-scores [46]. In Malawi continued LNS supplementation to women during pregnancy and the first 6 months postpartum and to their children thereafter had no impact on child anthropometry at 18 months [47].

As feeding practices are similar to those found by Patel in northern India [48], we hypothesise that the disappearance of anthropometric differences detected at birth in our study may be associated with late introduction and insufficient quality or quantity of complementary foods. A high burden of infection, especially environmental enteropathy [49], associated with poor sanitation and low coverage of toilets in the area might also explain the increase in undernutrition we found between birth and 9 months on average [50]. Heady and Hoddinott estimate that reduction in stunting over time in younger children (0–12 months) in Nepal is 18% attributable to improvements in sanitation, 29% to health care, 25% to increased wealth and 19% to maternal education. They hypothesise that most of the improvements in nutritional status in Nepalese children between 2001 and 2011 were driven by increases in birth size and less by improved postnatal nutrition trajectories [51]. Whilst our findings support their finding of poor postnatal growth, the association between birth size and pregnancy interventions had disappeared nine months later.

Increases in birthweight within 72 hours in the cash arm of our study were not large enough to reach significance but evidence from South American settings of large-scale cash transfer programmes showed increases in birthweight of 31g in Uruguay through the PANES programme [21] and of 127g in Mexico through the Oportunidades programme [20] as well as improvements in child length in the first 6 months, though not necessarily later in childhood [22, 23].

Our ‘dose response’ analysis raises some concern that children born to mothers who received more cash transfers in pregnancy were slightly smaller at endpoint. We suggest that this result is likely to arise from residual confounding whereby women who received more transfers differed in some way not captured in our model from those who received fewer.

The likely effect of the PLA plus food arm on institutional delivery and breastfeeding is interesting as the changes were unlikely to be associated with the food supplement itself. Enhanced participation in women’s groups brought about by provision of food transfers may have stimulated improved uptake of institutional delivery. It might also be the case that certain food arm clusters had higher institutional delivery rates prior to the intervention, though exploratory analyses did not show any significant difference between arms in pre-intervention and run-in periods of the study. During the main trial period, three clusters in the food arm had institutional delivery rates above 73%, which was not found elsewhere. It could be that the provision of care was better in these areas than elsewhere, though we do not have data on this. Any explanation of this finding can only be speculative.

The effects on colostrum feeding and timely initiation of breastfeeding may have been mediated by greater attendance at women’s groups in this arm, or by the increase in institutional deliveries because these practices would have been encouraged in health facilities. However, a similar trial of women’s groups practising PLA in Bangladesh showed a shorter time to initiation of breastfeeding without any associated increase in facility deliveries [52], as well as increased exclusive breastfeeding [30]. It may be that breastfeeding behaviour was also susceptible to change amongst those who delivered at home in our study population but we have not tested this.

The increased effect on birthweight of adding transfers of food or cash to a PLA women’s group intervention suggests that providing women with extra resources during pregnancy increases their access to and intake of nutritious food. It may be that the PLA was more effective in the cash and food arms because the transfers incentivised group attendance and pregnant women and their mothers-in-law were thereby motivated to improve the diets of pregnant women.

Our study raises questions as to why supplementation with fortified wheat-soy blended food was apparently more effective in increasing birthweight than cash transfers. Perhaps women adhered to consumption of Super Cereal better than they adhered to spending their cash upon similarly energy-dense food? However a qualitative evaluation indicated that decision-makers in the household were often persuaded to spend cash transfers on pregnant women’s food intake [40]. In addition, some families saved their cash transfers to comply with PLA recommendations for women to attend antenatal care and seek institutional delivery in order to secure access to private providers [40]. Super Cereal did not have resale value on the local market and was not a substitute for regular meals: it differed from usual staples, was sweetened, and was not preferred. Cash could be spent as the pregnant woman chose or used for regular household expenses, though many reported spending their transfers on increasing milk intake [40]. The food ration was sufficient to allow for considerable household sharing, whilst the cash transfer was sufficient to buy 0.5 litres of milk daily, which, if shared, may not have increased intake as much as the food supplement.

A sub-study on pregnant women’s diets found that, compared with the control arm, women in the PLA plus cash arm consumed more micronutrient-rich foods, especially dairy, whereas women in the PLA plus food arm may have been allocated slightly more energy [53]. This suggests that the food and cash were affecting dietary patterns in different ways. This sub-study was conducted towards the end of the trial, on a sub-sample of pregnant women in their third trimester, when respondents were eating relatively small amounts of super cereal per day. It seems that regular consumption of fortified balanced protein-energy super cereal (during the earlier time of the study when birthweight was being measured) was more effective at improving birthweight than regular consumption of extra milk, as occurred in the cash arm. In this context, gender disparity in earned cash income is a stronger predictor of intra-household calorie allocation than household food security, whereas household food security is a stronger predictor of micronutrient allocation [54]. Yet, intra-household allocation of dairy was more equitable in the PLA plus cash arm than the control, and intra-household allocation of energy was more equitable in the PLA plus food arm, suggesting that the additional dairy purchased with the cash and the additional energy from Super Cereal in these transfer arms were channelled to the pregnant women (51). A sub-study on the impact of trial interventions upon women’s agency found that the impact on birthweight was not mediated by an increase in women’s empowerment. The PLA intervention increased agency in the study population by enabling newly married women to attend group meetings in the community, a privilege normally reserved for their women 10–20 years older than them. However, we found no impact on agency in domains other than group participation including health behaviours [55]. It could be that PLA increased awareness about maternal health and nutrition amongst decision-makers in households and that this led to increased antenatal care seeking and better hygiene. This in turn may have improved pregnant women’s health and eating behaviours, but our response rates were too low to draw conclusions on this.

Future studies should compare the effect of cash and nutritious food transfers on birthweight and on longer-term growth and survival, at least to two years. Ideally they should test the effect of nutritional interventions throughout the ‘thousand days’ period from conception, through pregnancy, exclusive breastfeeding and complementary feeding to 2 years of age, starting at the pre-conception stage with newly weds or couples planning a family. The apparent graded effect on birthweight through PLA alone, PLA plus cash, and PLA plus food also suggests that further studies using PLA for nutrition behaviour change are warranted.

Compliance with our interventions in the study area was high, especially in the PLA + food and PLA + cash arms. Despite the limitations of our birthweight data, adding food supplements to a PLA intervention in pregnancy increased birthweight significantly. Increases in weight or length at birth were not sustained through the first 16 months of life, which suggests that pregnancy interventions alone are unlikely to have lasting impacts on child nutritional status in these settings. Investment in fortified food supplementation combined with PLA groups for pregnant women may increase birthweight. Although continued support for breastfeeding, complementary feeding, improved water, sanitation and hygiene and infection control is also essential to break the intergenerational cycle of undernutrition in South Asia, an improvement in birthweight is an important achievement in its own right, since birthweight is an indicator of fetal development and a predictor of chronic disease in later life (2, 4, 5).

## Conclusions

This study is the first to use women’s groups practicing Participatory Learning and Action (PLA) to influence birthweight as the primary outcome in a cluster-randomised controlled trial, and to compare the impact on birthweight of nutritious food and cash transfers combined with PLA. It is also the first to assess the effect of these pregnancy interventions on child nutritional status after birth and the first to assess impact of cash transfer using an RCT design. We confirm others’ findings that provision of food, a fortified balanced energy protein supplement in our case, in pregnancy can increase birthweight, but evidence for the impact of cash transfers (7.5 USD/mo) is less clear. PLA alone did not increase birthweight substantially and did not attain significance.

Provision of nutritious food supplements to pregnant women can increase the weight of babies at birth and may be more effective than provision of a modest amount of cash to buy additional food in pregnancy. However, in environments with very poor sanitation and hygiene, where infant and young child feeding behaviours are inadequate, gains in weight resulting from pregnancy interventions are not sustained, which suggests that nutrition interventions in pregnancy may need to continue beyond pregnancy until at least 2 years of age. Further studies comparing the effects of food and cash and assessing the impact of PLA on birthweight and child nutrition are needed, as are studies evaluating the long-term impacts on health of increases in birthweight.

## Supporting information

S1 Table Outcome measures and questionnaire tools used to capture them. (DOCX)

S2 Table Capture rates of different questionnaires in all cases, cases eligible for weight-for-age z-score analyses, and cases eligible for birthweight analyses, after exclusion of cases. (DOCX)

S3 Table Characteristics of those whose birthweights were and were not captured. (DOCX)

S4 Table Birth outcomes and mortality outcomes, by study arm. (DOCX)

S5 Table Analyses of interaction between multidimensional poverty groups and study interventions. (DOCX)

S1 File Low birth weight South Asia trial published protocol. (PDF)

S2 File Consort checklist of the low birth weight South Asia trial. (DOC)

S3 File Read me file about low birth weight South Asia trial dataset. (DOCX)

S4 File Low birth weight South Asia trial data use application form. (DOCX)
