# Supplementary material for: Impact on birth weight and child growth of Participatory Learning and Action women’s groups with and without transfers of food or cash during pregnancy: Findings of the low birth weight South Asia cluster-randomised controlled trial (LBWSAT) in Nepal
Source: PLoS One. 2018 May 9;13(5):e0194064. doi: 10.1371/journal.pone.0194064 (PMC5942768; doi:10.1371/journal.pone.0194064)
Supplement: S1 Table — (DOCX) [file pone.0194064.s001.docx]

**S1 Table. Outcome measures and questionnaire tools used to capture them.**

| **Outcome** | **Main surveillance tools** | | | | | |  | **Endpoint Nutrition Follow-up clinics** |
| --- | --- | --- | --- | --- | --- | --- | --- | --- |
|  | **Check suspected pregnancy** | **Socioeconomic** | **Early Pregnancy** | **Late Pregnancy** | **Delivery** | **Post Neonatal** | **Woman update from register** |  |
| **Primary outcomes** |  |  |  |  |  |  |  |  |
| Primary outcome: Birth weight, measured within 72 hours after birth. |  |  |  |  | ✓ |  |  |  |
| Second primary outcome: weight-for-age Z-score, measured at 0-24 months |  |  |  |  |  |  |  | ✓ |
| **Secondary outcomes** |  |  |  |  |  |  |  |  |
| Low birth weight (< 2500g) |  |  |  |  | ✓ |  |  |  |
| Birth weight measured within 10 days |  |  |  |  | ✓ |  |  |  |
| Weight for age Z-score taken within 42 days |  |  |  |  | ✓ | ✓ |  |  |
| Length measured within 10 days |  |  |  |  | ✓ | ✓ |  |  |
| Head circumference measured within 10 days |  |  |  |  | ✓ |  |  |  |
| Maternal weight or weight gain during pregnancy |  |  | ✓ | ✓ |  |  |  |  |
| Preterm delivery (based on LMP) | ✓ |  |  |  | ✓ |  |  | ✓ |
| Miscarriages |  |  |  |  |  |  | ✓ | ✓ |
| Stillbirths |  |  |  |  | ✓ |  | ✓ | ✓ |
| Neonatal mortality |  |  |  |  | ✓ | ✓ | ✓ | ✓ |
| Maternal morbidity during pregnancy |  |  | ✓ | ✓ | ✓ | ✓ |  |  |
| Neonatal morbidity |  |  |  |  |  | ✓ |  |  |
| Post neonatal mortality |  |  |  |  |  | ✓ |  | ✓ |
| Maternal deaths |  |  |  |  | ✓ | ✓ | ✓ | ✓ |
| Maternal eating behaviour in pregnancy (eating down, maternal 24-hour dietary diversity score in early and late pregnancy, consumption of key micronutrient-rich foods, number of eating occasions per day, observance of food taboos and fasting) |  |  | ✓ | ✓ |  |  |  |  |
| Length-for-age Z-score 0-24 months |  |  |  |  |  |  |  | ✓ |
| Weight -for-length Z-score 0-24 months |  |  |  |  |  |  |  | ✓ |
| Head circumference 0-24 months |  |  |  |  |  |  |  | ✓ |
| Maternal BMI (within 24 months of birth) |  |  |  |  |  |  |  | ✓ |
| Maternal Mid-upper Arm Circumference (MUAC) (within 24 months of birth) |  |  |  |  |  |  |  | ✓ |
| Child morbidity |  |  |  |  |  |  |  | ✓ |
| Child dietary diversity (after 6 months) and consumption of key foods (animal food, green leafy veg, fruits and veg) |  |  |  |  |  |  |  | ✓ |
| Early breastfeeding behaviour (colostrum discarding/ time of initiation) |  |  |  |  |  |  |  | ✓ |
| Infant feeding (exclusive breastfeeding, prolonged breastfeeding) |  |  |  |  |  |  |  | ✓ |
| Institutional delivery |  |  |  |  |  |  |  | ✓ |
