# Supplementary material for: Impact on birth weight and child growth of Participatory Learning and Action women’s groups with and without transfers of food or cash during pregnancy: Findings of the low birth weight South Asia cluster-randomised controlled trial (LBWSAT) in Nepal
Source: PLoS One. 2018 May 9;13(5):e0194064. doi: 10.1371/journal.pone.0194064 (PMC5942768; doi:10.1371/journal.pone.0194064)
Supplement: S2 Table — (DOCX) [file pone.0194064.s002.docx]

**S2 Table. Capture rates of different questionnaires in all cases, cases eligible for weight-for-age Z-score analyses and cases eligible for birth weight analyses, after exclusion of cases.**

| **Type of questionnaire** | **All cases before exclusions** | | | | | | **In WAZ trial after exclusions**^2^ | | **In BW trial after exclusions**^1^ | |
| --- | --- | --- | --- | --- | --- | --- | --- | --- | --- | --- |
|  | **Control** | **Women's group (PLA) only** | **PLA + Cash** | **PLA + Food** | **No of cases all arms** | ***% capture*** | **No of cases** | **% capture** | **No of cases** | **% capture** |
| Socioeconomic at enrolment | 4,775 | 4,825 | 6,067 | 5,480 | 21,147 | *84.3%* | 9,456 | 86.5% | 8,406 | 85.9% |
| Socioeconomic at endpoint | 435 | 650 | 945 | 1,047 | 3,077 | *12.3%* | 1,319 | 12.1% | 1,182 | 12.1% |
| *Total socioeconomic available* | 5,822 | 5,475 | 7,012 | 6,527 | 24,224 | *96.5%* | *10,775* | 98.5% | *9,588* | 98.0% |
| Early pregnancy | 674 | 611 | 1,484 | 1,189 | 3,958 | *15.8%* | 2,239 | 20.5% | 1,782 | 18.2% |
| Third trimester | 657 | 549 | 927 | 781 | 2,904 | *11.6%* | 1,899 | 17.4% | 1,801 | 18.4% |
| Delivery | 2,038 | 1,828 | 1,967 | 2,106 | 7,939 | *31.6%* | 4,500 | 41.1% | 4,577 | 46.8% |
| Post neonatal | 986 | 760 | 677 | 615 | 3,038 | *12.1%* | 2,189 | 20.0% | 2,227 | 22.8% |
| Maternal questionnaire at endpoint | 4,143 | 4,416 | 5,398 | 4,980 | 18,937 | *75.5%* | 9,512 | 87.0% | 8,566 | 87.5% |
| Child questionnaire at endpoint | 4,171 | 4,470 | 5,451 | 5,051 | 19,143 | *76.3%* | 9,520 | 87.1% | 8,579 | 87.7% |
| Birth weight within 72 hours | 760 | 747 | 878 | 1,097 | 3,482 | 13.9% | 2,111 | 19.3% | **2,147** | 21.9% |
| Weight-for-Age Z-score at endpoint | 3,667 | 3,889 | 4,658 | 4,410 | 16,624 | 66.3% | **9,242** | 84.5% | 8,046 | 82.2% |
| **TOTAL** |  |  |  |  | **25,092** |  | **10,936** |  | **9,786** |  |

^1.^ Excluding miscarriages/ terminations, stillbirths, multiple births, children whose mothers have died, stillbirths, neonatal deaths occurring before birth weight can be taken, births before start of interventions (13 Feb 2014) and during run-in period (13 Feb – 4 Jun 2014), infants born after closure of birth weight measurement (31 March 2015), infants born to in-migrating women and infants with congenital abnormalities

^2.^ Excluding all in (^1^) plus neonatal deaths after 72 hours and post-neonatal deaths but including infants born after closure of birth weight measurement (31 March 2015) and before start of endpoint nutrition ‘clinic’ data collection (20 Jun 2015)
