# Supplementary material for: Impact on birth weight and child growth of Participatory Learning and Action women’s groups with and without transfers of food or cash during pregnancy: Findings of the low birth weight South Asia cluster-randomised controlled trial (LBWSAT) in Nepal
Source: PLoS One. 2018 May 9;13(5):e0194064. doi: 10.1371/journal.pone.0194064 (PMC5942768; doi:10.1371/journal.pone.0194064)
Supplement: S3 Table — (DOCX) [file pone.0194064.s003.docx]

**S3 Table. Characteristics of those whose birthweights were and were not captured**

| **Characteristic** | **Birthweight not captured** | | **Birthweight captured** | | **Total** | | Odds of Birth-weight capture (OR) | *95 CI* | p |
| --- | --- | --- | --- | --- | --- | --- | --- | --- | --- |
|  | Freq | % | Freq | % | Freq | % |  |  |  |
| **Study arm woman enrolled in** *(N)* |  |  |  |  | *9786* |  |  |  |  |
| 0. Control | 1241 | 20.6 | 924 | 24.6 | 2165 | 22.1 | ref |  |  |
| 1. Women's group | 1361 | 22.6 | 837 | 22.2 | 2198 | 22.5 | 0.825 | *(0.449,1.516)* | 0.9276 |
| 2. Cash | 1760 | 29.2 | 956 | 25.4 | 2716 | 27.8 | 0.856 | *(0.467,1.569)* |  |
| 3. Food | 1662 | 27.6 | 1045 | 27.8 | 2707 | 27.7 | 0.927 | *(0.505,1.670)* |  |
| **Randomisation stratum** *(N)* |  |  |  |  | *9786* |  |  |  |  |
| small, inaccessible | 1099 | 18.2 | 893 | 23.7 | 1992 | 20.4 | ref |  |  |
| small, accessible | 1337 | 22.2 | 811 | 21.6 | 2148 | 21.9 | 0.742 | (0.408,1.349) | 0.1193 |
| large, inaccessible | 1760 | 29.2 | 1124 | 29.9 | 2884 | 29.5 | 0.823 | (0.454,1.493) |  |
| large, accessible | 1828 | 30.3 | 934 | 24.8 | 2762 | 28.2 | 0.588 | (0.323,1.067) |  |
| **Age of pregnant woman** *(N)* |  |  |  |  | *9786* |  |  |  |  |
| 1. 12-19 years | 2445 | 40.6 | 1332 | 35.4 | 3777 | 38.6 |  |  |  |
| 2. 20-24 years | 2308 | 38.3 | 1460 | 38.8 | 3768 | 38.5 |  |  |  |
| 3. 25-29 years | 982 | 16.3 | 712 | 18.9 | 1694 | 17.3 |  |  |  |
| 4. 30-46 years | 289 | 4.8 | 258 | 6.9 | 547 | 5.6 |  |  |  |
| (mean *sd*) | 21.51 | *4.45* | 22.14 | *4.74* | 21.75 | *4.57* | 1.033 | *(1.023,1.043)* | **<0.0001** |
| **Gravida (previous pregnancies)** *(N)* |  |  |  |  | *9588* |  |  |  |  |
| 0 | 1989 | 34.1 | 1110 | 29.6 | 3099 | 32.3 |  |  |  |
| 1 | 1675 | 28.7 | 990 | 26.4 | 2665 | 27.8 |  |  |  |
| 2 | 1184 | 20.3 | 817 | 21.8 | 2001 | 20.9 |  |  |  |
| 3 | 589 | 10.1 | 465 | 12.4 | 1054 | 11.0 |  |  |  |
| 4 or more | 398 | 6.8 | 371 | 9.9 | 769 | 8.0 |  |  |  |
| **Parity (previous births)** (mean *sd*) | 1.25 | *1.31* | 1.46 | *1.41* | 1.33 | *1.35* | 1.124 | *(1.087,1.162)* | **<0.0001** |
| **Mother's education** *(N)* |  |  |  |  | *9585* |  |  |  |  |
| 1. never went to school | 3701 | 63.5 | 2472 | 65.9 | 6173 | 64.4 | ref |  |  |
| 2. primary to lower secondary | 1211 | 20.8 | 737 | 19.6 | 1948 | 20.3 | 0.878 | *(0.783,0.984)* | **0.0024** |
| 3. secondary and above | 920 | 15.8 | 544 | 14.5 | 1464 | 15.3 | 0.817 | *(0.718,0.929)* |  |
| **Asset quintile**  *(N)* | Freq | % |  |  | *9496* |  |  |  |  |
| 1 | 1080 | 18.8 | 700 | 18.7 | 1780 | 18.7 |  |  |  |
| 2 | 1179 | 20.5 | 756 | 20.2 | 1935 | 20.4 |  |  |  |
| 3 | 1175 | 20.4 | 774 | 20.7 | 1949 | 20.5 |  |  |  |
| 4 | 1116 | 19.4 | 815 | 21.8 | 1931 | 20.3 |  |  |  |
| 5 | 1204 | 20.9 | 697 | 18.6 | 1901 | 20.0 |  |  |  |
| **1 SD in Asset score** (mean *sd*) | 0.04 | *1.02* | 0.00 | *0.98* | 0.02 | *1.01* | 0.961 | *(0.917,1.006)* | 0.0882 |
| **Husband migrated for labour** *(N)* |  |  |  |  | *8407* |  |  |  |  |
| Husband had not migrated | 3528 | *73.8* | 2691 | 74.2 | 6219 | 74.0 | ref |  |  |
| Husband migrated | 1250 | *26.2* | 938 | 25.8 | 2188 | 26.0 | 0.957 | (0.860,1.066) | 0.4259 |
| **Caste 3 groups** *(N)* |  |  |  |  | *9786* |  |  |  |  |
| Dalit/Muslim- disadvantaged | 2095 | *34.8* | 1285 | 34.2 | 3380 | 34.5 | ref |  |  |
| Janjati/Other Terai castes - middle | 2514 | *41.7* | 1693 | 45.0 | 4207 | 43.0 | 1.01 | *(0.910,1.119)* | 0.7206 |
| Yadav/Brahmin least disadvantaged | 1415 | *23.5* | 784 | 20.8 | 2199 | 22.5 | 0.96 | *(0.843,1.091)* |  |
| **Sex of baby** *(N)* |  |  |  |  | *8922* |  |  |  |  |
| Female | 2397 | *46.4* | 1811 | 48.2 | 4208 | 47.2 |  |  |  |
| Male | 2765 | *53.6* | 1949 | 51.8 | 4714 | 52.8 | 0.930 | (0.849,1.019) | 0.1193 |
| **Religion** *(N)* |  |  |  |  | *9785* |  |  |  |  |
| Muslim or other | 1008 | *16.7* | 472 | 12.5 | 1480 | 15.1 | ref |  |  |
| Hindu | 5015 | *83.3* | 3290 | 87.5 | 8305 | 84.9 | 1.298 | *(1.135,1.484)* | **0.0001** |
| **Mother's Height Category** *(N)* |  |  |  |  | *8687* |  |  |  |  |
| Not short stature >=145cm | 4350 | *85.0* | 3009 | 84.3 | 7359 | 84.7 |  |  |  |
| Short stature <145cm | 766 | *15.0* | 562 | 15.7 | 1328 | 15.3 |  |  |  |
| **Mother's height in cm** (mean *sd*) | 150.6 | *5.4* | 150.3 | *5.3* | 150.5 | *5.4* | 0.992 | *(0.984,1.001)* | 0.0857 |
