# Supplementary material for: Impact on birth weight and child growth of Participatory Learning and Action women’s groups with and without transfers of food or cash during pregnancy: Findings of the low birth weight South Asia cluster-randomised controlled trial (LBWSAT) in Nepal
Source: PLoS One. 2018 May 9;13(5):e0194064. doi: 10.1371/journal.pone.0194064 (PMC5942768; doi:10.1371/journal.pone.0194064)
Supplement: S4 Table — (DOCX) [file pone.0194064.s004.docx]

**S4 Table. Birth outcomes and mortality outcomes by study arm.**

|  | **0. Control** | | | **1. Women's group (PLA) only** | | | **2. PLA + Cash** | | | **3. PLA + Food** | | | **Total** | | |
| --- | --- | --- | --- | --- | --- | --- | --- | --- | --- | --- | --- | --- | --- | --- | --- |
| **Birth outcome** | Birth weight & WAZ^1^ | WAZ only^2^ | **All**^3^ | Birth weight & WAZ^1^ | WAZ only^2^ | **All**^3^ | Birth weight & WAZ^1^ | WAZ only^2^ | **All**^3^ | Birth weight & WAZ^1^ | WAZ only^2^ | **All**^3^ | Birth weight & WAZ^1^ | WAZ only^2^ | **All**^3^ |
| Miscarriage/abortion | 46 | 7 | **53** | 67 | 11 | **78** | 159 | 23 | **182** | 133 | 30 | **163** | 405 | 71 | **476** |
| Multiple Birth | 17 | 5 | **22** | 20 | 1 | **21** | 29 | 2 | **31** | 30 | 1 | **31** | 96 | 9 | **105** |
| **Mortality outcome of singleton /first baby of multiple births** |  |  |  |  |  |  |  |  |  |  |  |  |  |  |  |
| Stillbirth | 36 | 4 | **40** | 40 | 3 | **43** | 34 | 5 | **39** | 40 | 4 | **44** | 150 | 16 | **166** |
| Neonatal death within 72 hours | 20 | 0 | **20** | 15 | 2 | **17** | 11 | 1 | **12** | 18 | 1 | **19** | 64 | 4 | **68** |
| Neonatal death after 72 hours | 37 | 4 | **41** | 51 | 6 | **57** | 46 | 11 | **57** | 60 | 4 | **64** | 194 | 25 | **219** |
| *All neonatal deaths* | *57* | *4* | ***61*** | *66* | *8* | ***74*** | *57* | *12* | ***69*** | *78* | *5* | ***83*** | *258* | *29* | ***287*** |
| Post-neonatal death | 22 | 1 | **23** | 19 | 3 | **22** | 29 | 1 | **30** | 20 | 0 | **20** | 90 | 5 | **95** |
| **Deaths of mothers** |  |  |  |  |  |  |  |  |  |  |  |  |  |  |  |
| Pregnancy-related death in pregnancy or within 42 days of delivery | 0 | 0 | **0** | 1 | 0 | **1** | 3 | 0 | **3** | 1 | 0 | **1** | 5 | 0 | **5** |
| Death of mother 43 days to 1 year | 0 | 0 | **0** | 0 | 0 | **0** | 0 | 0 | **0** | 3 | 0 | **3** | 3 | 0 | **3** |
| Total permanent resident women delivering in each time period | 2,288 | 340 | **2,628** | 2,344 | 342 | **2,686** | 2,960 | 465 | **3,425** | 2,939 | 407 | **3,346** | 10,531 | 1,554 | **12,085** |

^1.^ Pregnancies of permanent resident trial participants who delivered or were due to deliver between 5 Jun 2014 and 31 March 2015

^2.^ Pregnancies of permanent resident trial participants who delivered or were due to deliver between 1 April 2015 and 19 Jun 2015

^3.^ All pregnancies of permanent residents who delivered or were due to deliver between 5 Jun 2015 and 19 Jun 2015
