# Supplementary material for: Impact on birth weight and child growth of Participatory Learning and Action women’s groups with and without transfers of food or cash during pregnancy: Findings of the low birth weight South Asia cluster-randomised controlled trial (LBWSAT) in Nepal
Source: PLoS One. 2018 May 9;13(5):e0194064. doi: 10.1371/journal.pone.0194064 (PMC5942768; doi:10.1371/journal.pone.0194064)
Supplement: S5 Table — (DOCX) [file pone.0194064.s005.docx]

**S5 Table. Analyses of interaction effects between multidimensional poverty groups and study interventions.**

|  | **Weight-for-age Z-score (no imputation)** | | **Weight-for-age Z-score with imputed covariates** | | **Length-for-age Z-score (no imputation)** | | **Length-for-age Z-score with imputed covariates** | |
| --- | --- | --- | --- | --- | --- | --- | --- | --- |
| **Covariate** | **Coeff.** | ***95% CI*** | **Coeff.** | **95% CI** | **Coeff.** | **95% CI** | **Coeff.** | **95% CI** |
| Middle | 0.077 | *(-0.041,0.195)* | 0.089 | (-0.026,0.205) | 0.111 | (-0.024,0.246) | 0.1258 | (-0.008,0.259) |
| Least Poor | 0.276 | ***(0.156,0.398)*** | **0.293** | **(0.177,0.410)** | **0.396** | **(0.257,0.535)** | **0.4081** | **(0.271,0.545)** |
| PLA only | -0.082 | *(-0.211,0.047)* | -0.044 | (-0.172,0.084) | -0.010 | (-0.170,0.151) | 0.0288 | (-0.132,0.189) |
| PLA + Cash | -0.079 | *(-0.205,0.048)* | -0.060 | (-0.185,0.066) | -0.002 | (-0.161,0.156) | 0.0124 | (-0.145,0.170) |
| PLA + Food | -0.099 | *(-0.225,0.027)* | -0.068 | (-0.192,0.056) | -0.114 | (-0.272,0.045) | -0.0567 | (-0.212,0.099) |
| Test of interaction between MPI thirds and PLA only intervention relative to control | p = | *0.6050* | p = | 0.8824 | p = | 0.6500 | p = | 0.5315 |
| Test of interaction between MPI thirds and PLA + cash intervention relative to control | p = | *0.5631* | p = | 0.6805 | p = | 0.6325 | p = | 0.1395 |
| Test of interaction between MPI thirds and PLA + food intervention relative to control | p = | *0.6840* | p = | 0.6298 | p = | 0.0857 | p = | 0.5803 |
