# Supplementary material for: Impact on birth weight and child growth of Participatory Learning and Action women’s groups with and without transfers of food or cash during pregnancy: Findings of the low birth weight South Asia cluster-randomised controlled trial (LBWSAT) in Nepal
Source: PLoS One. 2018 May 9;13(5):e0194064. doi: 10.1371/journal.pone.0194064 (PMC5942768; doi:10.1371/journal.pone.0194064)
Supplement: S3 File — (DOCX) [file pone.0194064.s008.docx]

**ReadMe file about LBWSAT trial dataset**

This archive contains files containing data from **The Low Birth Weight South Asia Trial (LBWSAT)** used to analyse trial results published in PLOS One in 2018

Trial registration: ISRCTN75964374 Funding code number: PO 5675

Primary funder: UKaid from Department for International Development South Asia Research Hub.

The following files have been archived:

| **File name** | **Description** |
| --- | --- |
| lbwsat_trial_dataset.dta | This is a stata.dta file generated in Stata 15 from the Low Birth Weight South Asia Trial conducted in the plains of Nepal between 2012 and 2015. The dataset contains 11240 cases (pregnant women and singleton children born of the study) and 178 variables. The data are numerical values or categorical variables that are numerically coded and labelled. All categorical variables have value labels except for VDC. This is the cluster variable from which value labels have been removed for confidentiality of study participants. |
| label_list_coding_lbwsat_trial_dataset.xlsx | This file contains the coding (value labels assigned) to each of the categorical variables in lbwsat_trial_dataset.dta |
| ProjectInformation.docx | This file contains an explanation of the cluster randomised controlled trial that generated the data in file lbwsat_trial_dataset.dta |
| DataDescription_Extended.docx | This file contains a description of the data in file lbwsat_trial_dataset.dta that were used to analyse primary and secondary outcomes from the Low Birth Weight South Asia Trial cluster randomised controlled trial. |
| LBWSAT_trial_dataset_codebook.xlsx | This file contains a description of all variables with variable name, variable label, value label and storage type in lbwsat_trial_dataset.dta, including explanations of how some variables have been calculated from others. |
| LBWSAT_Consent_form_en_preg&births.pdf | Consent form for participants in the trial |
| LBWSAT_info_sheet_en_menstrual_mon.pdf | Project information sheet given to participants in menstrual monitoring in all arms of the study. |
| LBWSAT_info_sheet_en_WGonly_areas.pdf | Project information sheet given to participants in the Participatory Learning and Action (PLA) only arm of the study. |
| LBWSAT_info_sheet_en_govprog_areas.pdf | Project information sheet given to participants in the control (government services only) arm of the study. |
| LBWSAT_info_sheet_en_FOOD_areas.pdf | Project information sheet given to participants in the PLA plus food transfer arm of the study. |
| LBWSAT_info_sheet_en_CASH_areas.pdf | Project information sheet given to participants in the PLA plus cash transfer arm of the study. |
| MIRA_LBWSATdata_sharing_form_blank | Data sharing agreement to be downloaded, filled and returned to [n.saville@ucl.ac.uk](mailto:n.saville@ucl.ac.uk) if seeking use of the data. |

Publications arising from this trial can also be found at:

Trial Protocol: <https://dx.doi.org/10.1186/s12884-016-1102-x>

Paper on electronic data collection: <https://doi.org/10.1080/16549716.2017.1330858>

Paper reporting impact of trial interventions on women’s agency:

<https://www.tandfonline.com/doi/full/10.1080/00220388.2018.1448069>

Trial results paper: <https://doi.org/10.1371/journal.pone.0194064>
