# Supplementary material for: Impact on birth weight and child growth of Participatory Learning and Action women’s groups with and without transfers of food or cash during pregnancy: Findings of the low birth weight South Asia cluster-randomised controlled trial (LBWSAT) in Nepal
Source: PLoS One. 2018 May 9;13(5):e0194064. doi: 10.1371/journal.pone.0194064 (PMC5942768; doi:10.1371/journal.pone.0194064)
Supplement: S4 File — (DOCX) [file pone.0194064.s009.docx]

**MIRA - UCL Low Birth Weight South Asia Trial Data Use Application**

**Applicant contact information**

| Name | Click here to enter text. |
| --- | --- |
| Title | Click here to enter text. |
| Address | Click here to enter text. |
| Country of residence | Click here to enter text. |
| Phone | Click here to enter text. |
| E-mail | Click here to enter text. |

**Research project description**

| Project title | Click here to enter text. |
| --- | --- |
| Project description (details of research questions to be answered and analyses planned) | Click here to enter text. |

**Data protection plan**

| Where do you plan to store the data? (this must be a password protected secure location) | Click here to enter text. |
| --- | --- |
| Who will be able to access the data? (each researcher using the data should fill a separate form) | Click here to enter text. |

**Publication plans**

| Proposed publication(s) | Click here to enter text. |
| --- | --- |
| Will any of the MIRA or UCL team members serve as co-author on these publications? (since so much work went into generating these data we request authorship of at least one representative each from UCL and MIRA on each paper). Yes UCL team member 🗹 Yes MIRA team member 🗹 | |
| If so, who? (delete as appropriate) | Representatives of MIRA: Dr. DS Manandhar, Mr. Bhim Shrestha  Principal investigators /Representatives of UCL: Dr. Naomi M. Saville, Prof. Anthony Costello |

I, the undersigned, affirm that data I have requested will be used and stored in the manner described in the research project description and data protection plan, and agreeing to maintain confidentiality of respondents.

| Signature of applicant | Date: |
| --- | --- |
| Name of applicant (printed): | |

We, the undersigned, as responsible persons for Mother and Infant Research Activities (MIRA) and University College London Institute for Global Health respectively, affirm that we agree to sharing of the above-mentioned data, contingent upon the data being used and stored in the manner described in the research project description and data protection plan with confidentiality of respondents ensured.

| Signature of MIRA executive director | Date: |
| --- | --- |
| Name: Dr. D.S. Manandhar | |
| Signature of Low Birth Weight South Asia Trial Principal Investigator | Date: |
| Name: Dr. Naomi M Saville | |

Please complete and return this form to:

Dr Naomi Saville, Senior Research Associate. Email: **Saville, Naomi <n.saville@ucl.ac.uk>**

Address: UCL Institute for Global Health, University College London, 30 Guilford Street, London WC1N 1EH and Dr. D.S. Manandhar, Executive Director, MIRA (Mother and Infant Research Activities), Thapathali, GPO Box 921, Kathmandu, Nepal. Email: **D S Manandhar <dsm@mira.org.np>**
